# Evaluation of the epidemiological and economic impact of the ADLIFE intervention on medium- to long-term in patients with advanced chronic disease

**DOI:** 10.3389/fpubh.2025.1682492

**Published:** 2025-11-26

**Authors:** Igor Larrañaga, Javier Mar, Ania Gorostiza, Borja García-Lorenzo, Beñat Zubeltzu, Remedios Vega, Rachelle Kaye, Gil Levy, Olga Vishnevetsky, Rikke Lyngholm Christensen, Anne Dichmann Sorknæs, Natassia Garton, Anne Swoboda, Fritz Arndt, Lisa McCann, Roma Maguire, Morven Miller, Gokce B. Laleci Erturkmen, Mustafa Yuksel, Theodoros N. Arvanitis, Chao Tong, Jose I. Aznar-Baranda, Jessica Caballero, Nerea González, Juan de la Torre, Dolores Verdoy, Esteban de Manuel Keenoy, Ane Fullaondo, ADLIFE Consortium, Ana Ortega-Gil, Ángel Moro, Itxaso Alayo, Urko Aguirre, Bárbara López, Dipak Kalra, Oliver Gröne, Janika Blömeke-Cammin, Mikael Lilja, Tim Robbins, Natassia K. Juul, Alec Morton, Konstantinos Koutsouradis, Bunyamin Sarigul, Gokhan Yilmaz, Omid Pournik, Shramika Panchal, Ashley Peake

**Affiliations:** 1 Biosistemak Institute for Health Systems Research, Bilbao, Spain; 2 Network for Research on Chronicity, Primary Care and Health Promotion (RICAPPS), Bilbao, Spain; 3 Research Unit, Debagoiena Integrated Healthcare Organisation, Osakidetza-Basque Health Service, Arrasate-Mondragón, Spain; 4 Donostialdea Integrated Healthcare Organisation, Osakidetza-Basque Health Service, Donostia-San Sebastián, Spain; 5 Tolosaldea Integrated Healthcare Organisation, Osakidetza-Basque Health Service, Tolosa, Spain; 6 Assuta Medical Centre Ashdod, Ashdod, Israel; 7 Centre for Innovative Medical Technology (CIMT), Odense University Hospital, Odense, Denmark; 8 Emergency & Medical Department, Svendborg Hospital, Odense University Hospital & University of Southern Denmark, Svendborg, Denmark; 9 University Hospitals Coventry & Warwickshire, NHS Trust, Coventry, United Kingdom; 10 Gesunder Werra-Meißner-Kreis GmbH, Eschwege, Germany; 11 Department of Computing and Information Sciences, University of Strathclyde, Glasgow, United Kingdom; 12 SRDC, Ankara, Türkiye; 13 Department of Electronic, Electrical and Systems Engineering, University of Birmingham, Birmingham, United Kingdom; 14 NTT Data Spain, Bilbao, Spain; 15 Research Unit, Galdakao-Usansolo University Hospital, Barrualde-Galdakao Integrated Healthcare Organisation, Osakidetza-Basque Health Service, Galdakao, Spain

**Keywords:** advanced chronic disease, digital health, integrated care, personalised care plan, simulation model, discrete event simulation, economic evaluation, budget impact analysis

## Abstract

**Introduction:**

Patients with advanced chronic disease (ACD) experience transitions in their clinical stability, leading to increased healthcare resource use and costs. EU-funded ADLIFE digital intervention aimed to ensure their quality of life through individualised care plans, clinical decision-making support, and patient empowerment. This study assessed the impact and sustainability of ADLIFE.

**Materials and methods:**

Target population included patients aged ≥55 years with heart failure (HF) and/or chronic obstructive pulmonary disease (COPD). First, a discrete event simulation (DES) model was developed using data from Osakidetza-Basque Health Service to represent the natural history of the disease. Second, ADLIFE intervention was implemented in four pilot sites: Spain, England, Israel and Denmark. Intervention effect was quantified by comparing resource use between control and intervention groups. Finally, a budget impact analysis (BIA) was conducted by extrapolating the burden of the disease to 2030 under two scenarios: conventional and ADLIFE.

**Results:**

ADFLIFE intervention involved 370 patients (185 intervention, 185 control). Emergency visits and consultations with primary care professionals decreased significantly, while specialist consultations increased. Depending on the pilot site, projections estimated that ACD prevalence will increase by 37–50% by 2030, increasing associated costs. Under the ADLIFE scenario, the burden of the disease could be reduced by 1–2%, resulting in cumulative savings of €4–58 million.

**Discussion:**

Projections indicated a major challenge ahead due to a rise in ACD prevalence, highlighting the need for timely and effective healthcare responses. ADLIFE improved patient care and resource management, and its adoption could help reduce the disease burden and generate sustained long-term savings.

## Introduction

The increasing prevalence of chronic diseases, mainly due to an aging population, has led to a profound change in the healthcare paradigm (1, 2). The accumulation of multiple chronic conditions and polypharmacy (3–5), has shifted the focus from healthcare organisations mainly concerned with treating acute problems to those emphasising a continuum-of-care approach (6, 7).

In this sense, patients with advanced chronic disease (ACD) entail complex management challenges, as they often experience temporary or permanent functional decline, significantly impacting their independence and quality of life (8–10). Consequently, the natural history of the disease in these patients is characterised by frequent transitions between stable and unstable states over time (11–13). During stable phases, patients are typically managed at home under the supervision of primary care (PC) professionals. However, episodes of decompensation often require more specialised care, frequently leading to hospital care (HC) referral and resulting in increased resource utilisation and healthcare costs (11, 14, 15). Moreover, concerns persist that healthcare services organised by medical specialities still fail to adequately meet the needs of this growing population (16, 17).

The digitisation of health care has given birth to numerous tools and resources that improve healthcare services, including solutions that make health information more accessible to patients while keeping their data secure (18, 19). Thanks to information and communication technologies (ICT), healthcare providers have more alternatives at their disposal and patients receive better care and more accessible treatment (20). In the same way, the development of electronic health records (EHR) has facilitated the creation of databases that contain clinical data linked to administrative data, enabling the recording of all the interactions and resource use that patients have in the healthcare system (21). This opens the door to the implementation and assessment of digital-based interventions designed to enhance the care provided to patients (22–24). However, the economic evaluation of these technologies remains essential to support evidence-based decision-making (25, 26), with particular attention needed on the long-term impact and feasibility of these new solutions, an aspect that is often overlooked (21, 27, 28).

Simulation models can be effectively used to estimate the economic impact and long-term outcomes in such cases (29). These models are simplified representations of reality that capture its essential properties and relationships (30). They can provide insights into the behaviour of the system under study before real-life testing, as far as they mathematically simulate a real-life situation using simulation software (31). Since different predictions can be generated by altering input parameters of the model, simulation modelling serves as a tool to virtually explore and assess different scenarios (32, 33).

In this context, the EU-funded project ‘Integrated Personalized Care for Patients with Advanced Chronic Diseases to Improve Health and Quality of Life’ (ADLIFE) was designed to respond to the needs of older adults living with ACD through a digitally supported intervention (34). The aim was to help patients maintain their independence and quality of life by slowing functional decline and promoting the efficient use of healthcare resources. The digital solution developed was based on the interdisciplinary management of individualised care plans, support for clinical decision-making, and patient empowerment. The project involved six European countries, with the intervention implemented at pilot sites in four of them, where its effectiveness, implementation and technology acceptance, and socio-economic impact were assessed.

The present study specifically focuses on the socio-economic impact assessment, aiming to develop a simulation model that reflects the natural history of patients with ACD, and evaluates the impact and sustainability of the ADLIFE digital intervention within a healthcare system over the medium- to long-term.

## Materials and methods

**Figure 1:**
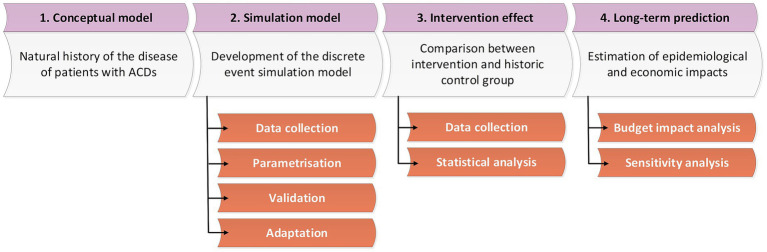
Main steps in the epidemiological and economic impact evaluation of the ADLIFE intervention.

The study was developed from the healthcare perspective and consisted of the comparison of two scenarios: the conventional scenario, which reflects the current standard of care, and the ADLIFE scenario, which represents a digitally enabled, integrated, and personalised model of care. First, a conceptual model representing the natural history of patients with ACD was defined. Second, a simulation model was developed, validated, and adapted to the specific context of each pilot site. Third, the ADLIFE intervention was implemented in the pilot sites, and its effect was quantified by measuring the change in resource use between control and intervention groups; these findings were then incorporated into the simulation model. Finally, medium- to long-term impacts were estimated, extrapolating the disease burden projected by the simulation models under both the conventional and ADLIFE scenarios. Figure 1 presents an overview of the main steps involved in the methodological approach adopted in this study.

### Target population

The target population consisted of patients aged 55 or older with severe heart failure (HF)—defined as NYHA class III–IV and/or ACCF/AHA stage C–D—and/or or severe chronic obstructive pulmonary disease (COPD)—defined as GOLD grade >2 (FEV1 <50%), mMRC grade ≥2, CAT score ≥10, and/or home oxygen use. Comorbidity criteria were defined using codes from the tenth revision of the international classifications of diseases (ICD-10). For HF I50.*, I11.0, I13.0, I13.2, I13.9 codes were used, while for COPD J44.* codes. Presence of active malignant neoplastic disease and/or inclusion in the active list of transplantation were considered as exclusion criteria. For malignant neoplastic disease C00.*–C97.* codes were used.

### Intervention

The ADLIFE intervention was designed to impact three key stakeholders: patients, informal caregivers, and healthcare professionals. The focus was on slowing patients’ functional decline to ensure their quality of life and independence, while optimising the use of healthcare resources. To achieve this, the ADLIFE toolbox was a digital solution comprising three ICT components: (1) a platform for the interdisciplinary management of individualised care plans, (2) a service designed to support clinical decision-making through the application of evidence-based clinical guidelines, and (3) a platform for patient empowerment (34). Personalised care plans for patients were created and managed in the personalised care plan management platform (PCPMP) by healthcare professionals, with clinical decision support services providing assistance in accordance with best clinical evidence. The approach facilitated coordination among different disciplines, as well as with patients and caregivers, ensuring the integration of services and supporting healthcare providers in making safe, accurate, standardised, and up-to-date decisions. PCPMP was integrated with pilot site ICT systems to generate care plans based on patients’ most recent clinical information. Patients and caregivers used the patient empowerment platform (PEP), which presented personalised goals, activities, and educational materials, collected observations and questionnaire responses, and delivered real-time interventions tailored to the patient’s lifestyle. The tool engaged patients and caregivers in self-managing their conditions, enhancing independence and autonomy, supporting adherence to treatment and care plans, and fostering shared decision-making.

Further details on the study design, recruitment process and analysis developed were described in the ADLIFE study protocol (34), while deviations from the protocol were addressed in the publicly available project documentation (35–37). As described in the protocol, using a mixed-methods approach, ADLIFE aimed to provide robust scientific evidence on the effectiveness assessment, implementation and technology acceptance assessment, and socio-economic impact assessment of the intervention, with this paper presenting the development and results of the latter. Six different pilot sites across six countries participated in the project: Basque Country (Spain), Coventry-Warwickshire (England), Ashdod (Israel), Syddanmark (Denmark), Werra-Meißner (Germany), and Lanarkshire (Scotland). Although all pilot sites participated in the project, the intervention was implemented between 2023 and 2024 only in the first four (37). The target sample size was set at 148 intervention and 148 control patients per site, amounting to a total of 1,184 participants (35). All participating patients signed informed consent, and the follow-up period was at least 3 months for all cases (36).

### Conceptual model

**Figure 2:**
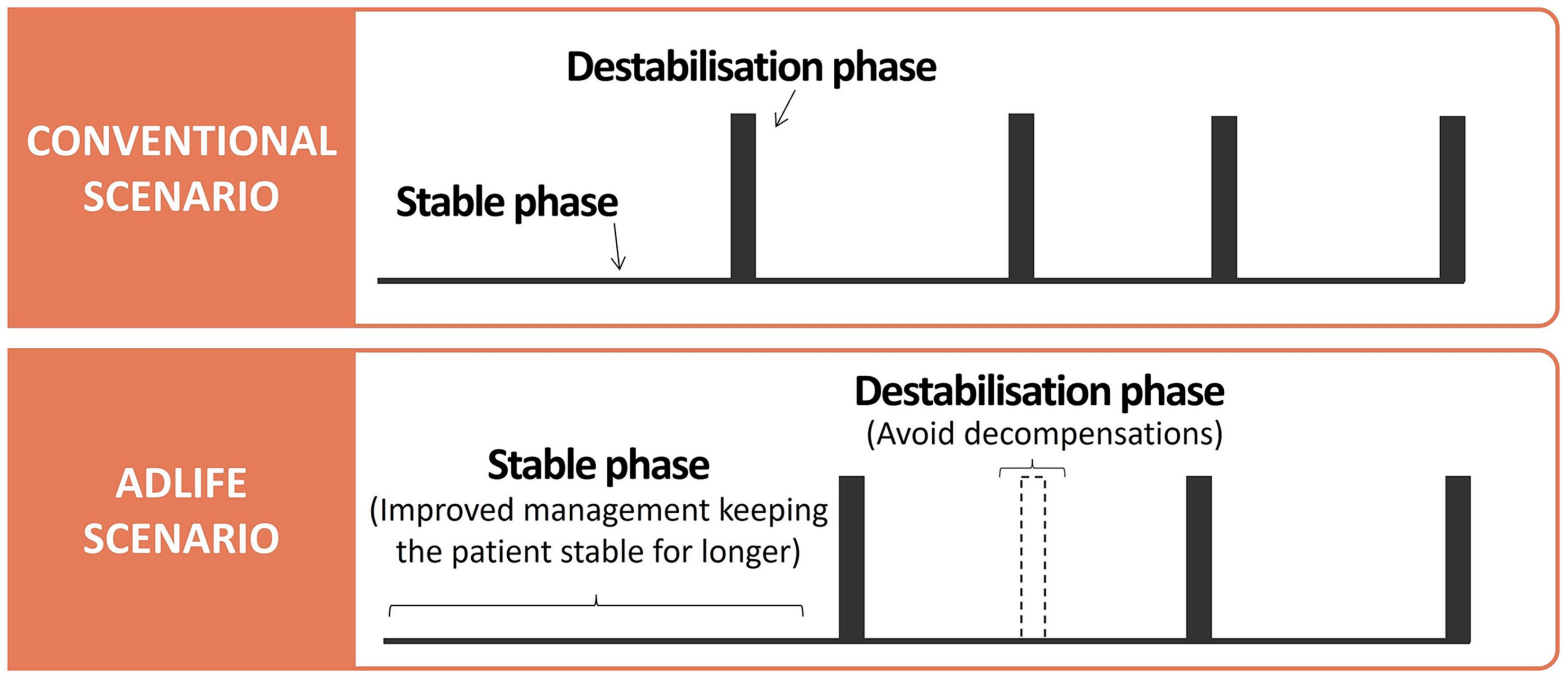
Conceptual differences in the progression of the natural history of the disease in patients with advanced chronic disease (ACD) between conventional and ADLIFE scenarios.

**Figure 3:**
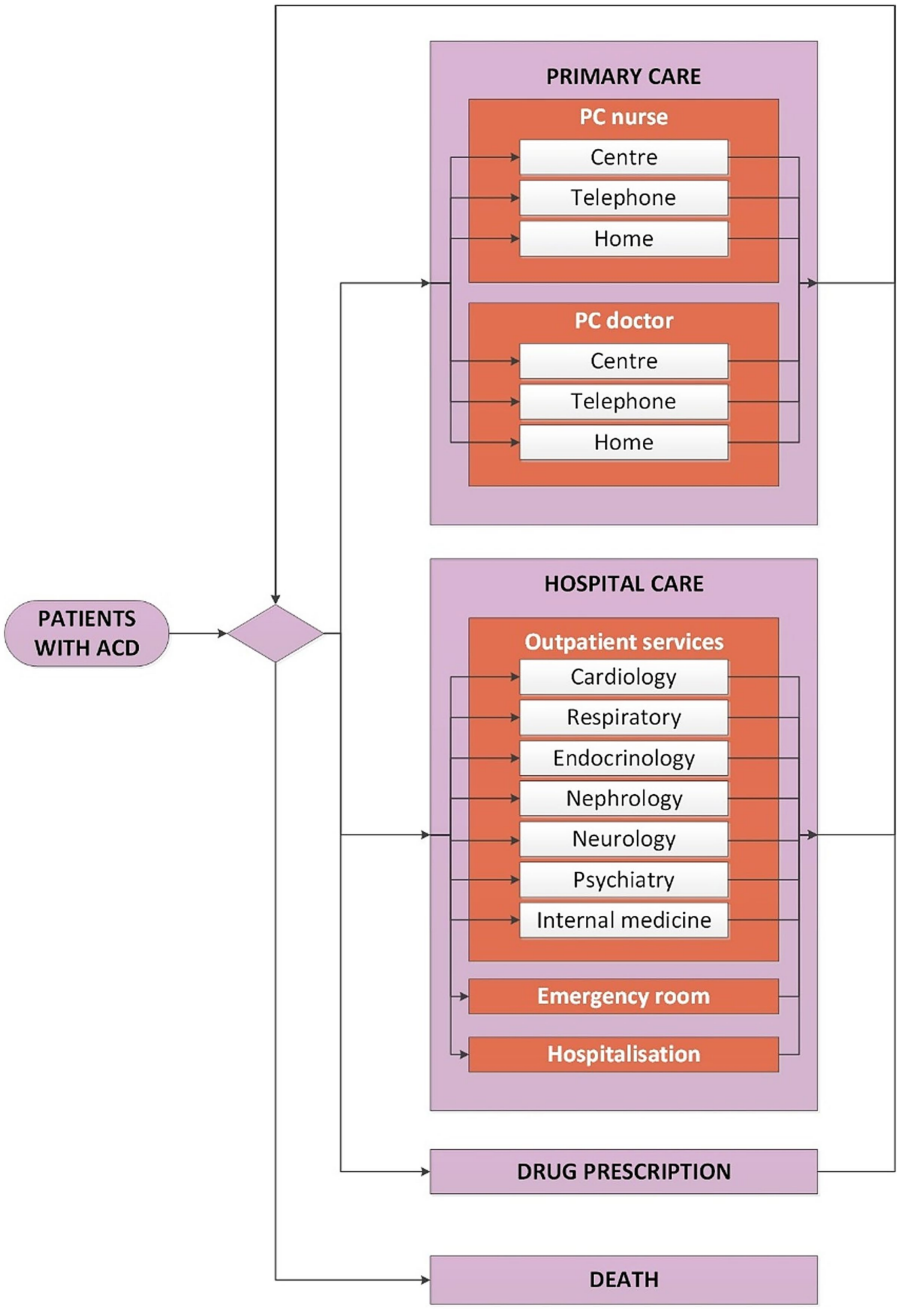
Conceptual model of the natural history of the disease in patients with advanced chronic disease (ACD).

The conceptual model used in ADLIFE was designed to represent the natural history of the disease as a dynamic process, characterised by frequent transitions into states of decompensation over time (11). To capture this, the disease trajectory was divided into stable and destabilisation as illustrated in Figure 2. The conceptual model encompassed all potential care pathways and contacts with the healthcare system that patients might experience throughout the course of the disease, as shown in Figure 3. During the stable phase, patients are primarily managed by PC professionals. In this phase, contacts with PC nurses and doctors, whether at healthcare centres, at home, or via telephone, were considered. During the destabilisation phase, patients require additional care and are typically referred to HC. In this phase, contacts with outpatient services (cardiology, respiratory, endocrinology, nephrology, neurology, psychiatry, and internal medicine), emergency room, and hospital admissions were considered. Throughout the entire process, drug consumption and mortality were also taken into account. The underlying hypothesis was that a patient-centred approach, focused on individualised care and early detection, would help manage and reduce destabilisation episodes, thereby decreasing the reliance on costly hospital resources such as emergency room visits and hospitalisations.

### Simulation model

A dynamic multi-cohort simulation model was developed using Arena software to represent the natural history of patients with ACD, based on the previously defined conceptual model (38, 39). For the development, the discrete event simulation (DES) technique was used, a flexible modelling method that can represent complex behaviours and interactions between different individuals, levels and environments (25, 31). DES reproduces the conceptual model by incorporating entities into a mathematical system and assigning them specific attributes or features. These entities represent individuals within a population, and by accounting for their entire journey through the system, the model generates outputs that help understand system behaviour and address research questions. The development process involved data collection, parameterisation, validation, and adaptation.

#### Data collection

Healthcare data needed to calculate the simulation parameters and populate the model were sourced from Osakidetza-Basque Health Service’s anonymised corporative databases. The information included patient-level demographic, epidemiological, and resource use data collected from 2012 to 2019. The cut-off was set in 2019 to avoid the impact of the COVID-19 pandemic on the resource use profile. Demographic data was composed by age, sex, Charlson index, ICD-10 diagnosis codes, date of diagnoses, and mortality. A descriptive analysis of the demographic variables is provided in the Supplementary Table S1. Regarding epidemiological data, prevalence and incidence of the disease were obtained by sex and age group. The resource use data included all contacts with healthcare resources identified in the conceptual model (Figure 3) for both PC and HC, as well as drug prescriptions.

Information on the unit cost of different healthcare resources was obtained directly from each pilot site respective health systems, except for Denmark, where the diagnosis related group rates was used (40). For all the pilot sites, unit costs were retrieved in euros (EUR, €). Details of the unit costs used for each pilot site are provided in the Supplementary Table S2.

Information on population figures and projections was collected from the official national statistical institutes of each pilot site region (41–46). Further details are available in the Supplementary Tables S3–S8.

#### Parametrisation

**Figure 4:**
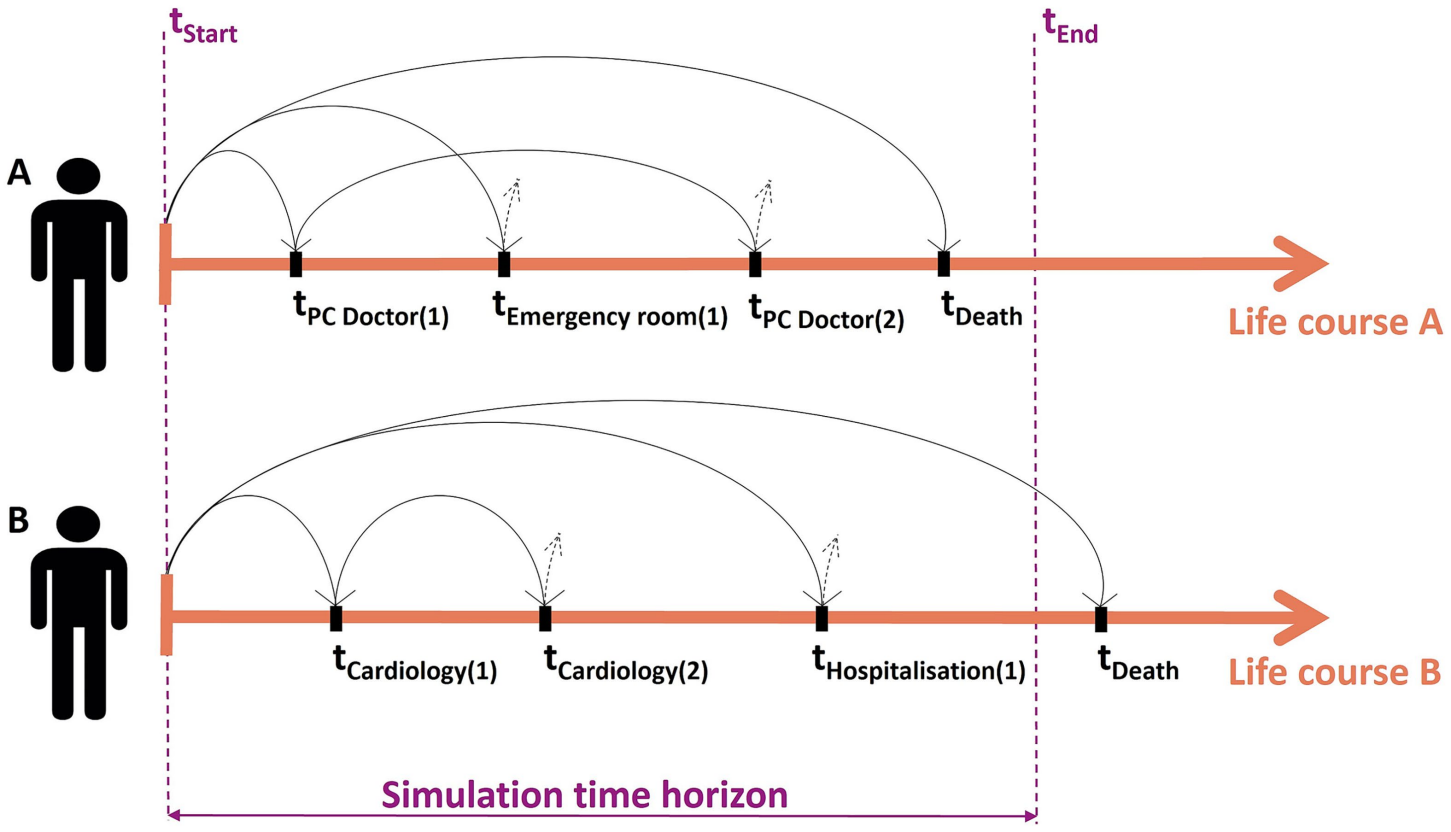
Rationale of the discrete event simulation (DES) model illustrated with two fictitious patients.

DES models require considering time in an explicit way. The rationale is that the natural history of the disease is converted into events that can occur in the life course of an individual and the time until those events is calculated as illustrated in Figure 4. In this study, the events corresponded to all interactions with healthcare identified in the conceptual model (Figure 3), and they were treated as competing risks. For every individual, a list of potential future events was generated based on their personal attributes and the competing risks. The next event to occur was identified as the one with the shortest time to occurrence. After an event occurred, the list of remaining events was updated, and the next event was again selected according to which was the closest in time. This process continued iteratively until the individual either died or the simulation time horizon ended. Individuals who remained alive or event-free at the end of the study period were considered survivors and treated as censored data. To obtain the simulation parameters required to model this process, all statistical analyses were conducted using Stata (version 14) or R (version 4.0.1).

On the one hand, the prevalence and incidence of patients with ACD were modelled by sex and age group. Patients eligible for ADLIFE before 2012 formed the prevalent cohort and entered the model at the simulation start. Those eligible afterward comprised incident cohorts, annually introduced by extrapolating incidence until 2019 based on population forecasts. Prevalent and incident cohorts derived from the Osakidetza-Basque Health Service databases are available in the Supplementary Table S9.

On the other hand, upon entering the model each individual was assigned a set of attributes (sex, age group, diseases, Charlson group and drug prescription cost) along with random values uniformly distributed between 0 and 1, ensuring variability in life trajectories even among individuals with the same characteristics. Logistic regressions were used to assign HF and/or COPD according to sex and age group, accounting for dependencies among chronic diseases (47). Similarly, logistic regressions determined Charlson group assignment according to age, sex and diseases. A linear regression with logarithmic transformation was used to estimate the drug prescription cost according to sex, age group, diseases and Charlson group. Parameters of the regression models used to define patient input characteristics are provided in the Supplementary Table S10.

Finally, time-to-event functions for the different competing risks were obtained developing a parametric survival analysis of the data. In the analysis different distributions were tested as survival functions: Gompertz, Weibull, exponential, lognormal, log-logistic and generalised gamma. All functions were adjusted by sex, age group, diseases and Charlson group. The type of function that best fit with the observed data was selected using the Akaike information criterion (AIC) (48, 49). Each function also included a hazard ratio (HR), which was used to incorporate the effect of the intervention into the model (50). Parameters of all time-to-event functions, as well as the expressions of these functions according to their underlying distributions, are accessible in the Supplementary Tables S11–S13 and Technical Note S1.

#### Validation

Once constructed, the model was validated comparing the simulated event rates with the observed ones from the year 2012 to 2019. The objective was to assure that the simulation model properly reproduced the conventional epidemiological scenario (51). For that purpose, a goodness-of-fit test was conducted with the following statistics (52): the correlation coefficient (R), normalised mean square error (NMSE), fractional bias (FB), fractional variance (FV) and the fraction of predictions within a factor of two (FAC2). To validate a model, the correlation coefficient and the factor of two must be higher than 0.8, the normalised mean squared error must be lower than 0.5 and the fractional bias and the fractional variance must be between −0.5 and 0.5. The goodness-of-fit test metrics obtained are provided in the Supplementary Tables S14–S16.

#### Adaptation

To ensure the model’s applicability across different pilot sites, unit costs and population projections were adjusted for each site to reflect local conditions. These adjustments allowed the model to be applied in diverse contexts and to estimate the evolution of disease burden over time for all of them. As the ADLIFE intervention was implemented between 2023 and 2024, the model was adapted accordingly to align with this timeframe and to generate results starting from that period.

First, unit costs were obtained and adjusted at each pilot site for the year 2023 to account for the specific economic context, reflecting differences in healthcare system structures, labour costs, medical supply prices, and facility overheads. These adjustments enhanced the accuracy of healthcare expenditure estimates across pilot sites. Detailed information is available in the Supplementary Table S2.

Second, site-specific population projections were incorporated to estimate trends in the prevalence and incidence of the ACD population from 2023 to 2030, accounting for local demographics such as population structure, birth and mortality rates, and migration. This approach enabled more accurate estimates of future disease burden at each pilot site by considering local demographic trends and population aging. The resulting prevalence and incidence projections are available in the Supplementary Tables S17–S22.

### Intervention effect

The effect of the ADLIFE intervention was assessed by measuring changes in the resource use profiles of participating patients compared to a historical control group. This effect was incorporated into the simulation model as HR, enabling differentiation between the conventional and ADLIFE scenarios. The HRs of resources that showed statistically significant differences were applied to the time-to-event functions (50).

#### Data collection

Information was obtained from healthcare databases based on pilot site experiences in Spain, England, Denmark, and Israel. Patients in the intervention group signed informed consent, but data on the historical control group were obtained retrospectively in an anonymised way. Propensity score matching was used to select control group patients by pairing each treated unit with a similar non-treated unit, ensuring comparability (53). One-to-one matching was applied to minimise bias (54).

All data were gathered using a data collection template specifically developed for the project, ensuring consistency and uniformity throughout the process (34). Demographic data included age, sex, diseases, Charlson index, mortality and follow-up. Resource use data included all contacts with healthcare resources identified in the conceptual model (Figure 3).

#### Statistical analysis

All the statistical analyses were performed using the free statistical software R (version 4.0.1) with a confidence level of 95%.

First, demographic and clinical differences between groups were analysed to ensure comparability. Healthcare resource use was also observed to address differences in resource consumption patterns. Fisher’s exact test was applied for categorical variables with two categories and expected frequencies less than or equal to 5, while the chi-square test was used in other cases. For continuous variables with a normal distribution, group means were compared using the Student’s t-test. Details of the descriptive analysis are presented in the Supplementary Tables S23, S24.

Second, adjusted regression models were used to assess the effect of the ADLIFE intervention. Logistic regressions were applied for dichotomous variables such as mortality. For resource use, the intervention effect was assessed using generalized linear models (GLM) (55). Given the nature of health services data, which often exhibit zero-inflated counts, negative binomial regression models were employed, whose results are additionally analogous in interpretation to HRs. A likelihood ratio test indicated that, due to the presence of overdispersion, the negative binomial regression model provided a better fit for the health services count data than the Poisson regression model. For hospital stays, only patients with any hospitalisation were included in the analysis. In the case of emergency room visits, since they were the primary outcome of the study (34), special consideration was required and they were analysed elsewhere using hurdle models (36). Hurdle models were developed to address excess zeros in count data when standard models such as Poisson or negative binomial are not optimal (56, 57). All models were adjusted by sex, age group, diseases and Charlson group. The time of follow-up also was included in the models as offset. The use of Cox regression models was discarded due to the nature of the data—count data—and the main purpose of the analysis, which was to assess the effectiveness of the intervention with respect to resource use. Consequently, HRs were derived from the outcomes of the regression models for subsequent incorporation into the simulation model (58, 59). The complete adjusted regression models, as well as the method used to approximate the outcomes to HR are detailed in the Supplementary Tables S25, S26 and Technical Note S2.

### Medium- to long-term impacts

The medium- to long-term epidemiological and economic impacts were estimated by running the simulation model. At this stage, the model was able to clone the target population to represent two alternative scenarios—conventional and ADLIFE—and generate outputs for both. Since the model replicated the random numbers assigned to each patient, both clones operated under identical conditions, with the only difference being the effect of the ADLIFE intervention.

#### Budget impact analysis

The evolution of the ACD population and their healthcare resource use were projected over time for both the conventional and ADLIFE scenarios (60). These projections were made from 2023 to 2030 including the impact of an aging population. The cost of the disease was calculated by multiplying the resource use rates predicted by the model with the unit costs collected from each pilot site. Consequently, the evolution of the burden of disease was estimated for both scenarios. The changes in healthcare expenditures resulting from the implementation of the ADLIFE intervention were assessed through a budget impact analysis (BIA) (29). The BIA estimates the financial consequences of adoption and diffusion of new healthcare interventions (61). Further details are provided in the Supplementary Tables S27–S38.

#### Sensitivity analysis

A sensitivity analysis was conducted to explore the performance of alternative scenarios and assess the robustness of the results. Structural assumptions within the model were tested to verify the long-term sustainability of the ADLIFE intervention. Since the simulation model was developed using actual data from the Osakidetza-Basque Health Service as its foundation, the scenario analysis was conducted exclusively for this case. The base case scenario was defined using the estimated intervention effect derived from pilot site experiences. Variations of this base case were explored by reducing the beneficial effects and increasing the detrimental effects of the intervention, as well as by modifying key assumptions such as patient numbers, resource utilisation rates, and unit costs. An additional scenario was also examined using data from the literature, based on effectiveness outcomes observed in the C3-CLOUD project (22, 62), a previous European initiative that implemented a similar intervention in a comparable but less severe population. The intervention effects obtained in the C3-CLOUD project are presented in the Supplementary Table S39, together with details of the scenario analysis (Supplementary Tables S40–S50).

## Results

During the development of the simulation model, data from the Osakidetza-Basque Health Service databases identified 104,500 patients between 2012 and 2019, as shown in Supplementary Table S1. This population had a mean age of approximately 76 years and higher proportion of men (53%). The goodness-of-fit test results, presented in Supplementary Tables S14–S16, indicate that the simulation model was properly validated and accurately reproduced conventional epidemiological scenario.

To assess the effect of the ADLIFE intervention, 370 patients were included in the trial, evenly distributed between the intervention (*n* = 185) and control (*n* = 185) groups, as shown in Table 1. The number of patients recruited in Spain, England, Israel, and Denmark was 46, 24, 148, and 152, respectively, such that the target sample size of 148 intervention and 148 control patients per site was not reached. The intervention group had an average follow-up duration of 7.5 months. There were no significant differences between the groups in terms of sex, age, comorbidities or Charlson index, confirming their comparability. The average age in both groups was around 71 years, with a higher proportion of male participants (53%). The intervention showed a significant reduction in in-person consultations at healthcare centre, with a 51% decrease for PC doctors. Additionally, telephone consultations decreased by 40% for PC doctors and by 56% for PC nurses. The probability of having an emergency room visit at the hospital in the intervention group was half that of the control group. Conversely, outpatient visits increased by 31%. No statistically significant differences were found in the use of other healthcare resources, including hospitalisations, nor in the probability of death. The main effects of the ADLIFE intervention are presented in Table 2, while the complete models can be found in Supplementary Tables S25, S26.

**Table 1:** Descriptive analysis of the ADLIFE sample comparing baseline characteristics between intervention and control groups.

Variable		Total (*N* = 370)		Intervention (*N* = 185)		Control (*N* = 185)		*p*-value^a^
		*n*	% (SE) or mean (SD)	*n*	% (SE) or mean (SD)	*n*	% (SE) or mean (SD)	
Sex	Women	175	47.3 (2.6)	87	47.0 (3.7)	88	47.6 (3.7)	1.00
	Men	195	52.7 (2.6)	98	53.0 (3.7)	97	52.4 (3.7)	
Age	Mean		71.4 (8.3)		71.1 (8.3)		71.7 (8.2)	0.49
	55–59 years	29	7.8 (1.4)	15	8.1 (2.0)	14	7.6 (1.9)	0.64
	60–64 years	40	10.8 (1.6)	20	10.8 (2.3)	20	10.8 (2.3)	
	65–69 years	79	21.4 (2.1)	45	24.3 (3.2)	34	18.4 (2.8)	
	70–74 years	73	19.7 (2.1)	31	16.8 (2.7)	42	22.7 (3.1)	
	75–79 years	78	21.1 (2.1)	42	22.7 (3.1)	36	19.5 (2.9)	
	80–84 years	46	12.4 (1.7)	19	10.3 (2.2)	27	14.6 (2.6)	
	85–89 years	18	4.9 (1.1)	9	4.9 (1.6)	9	4.9 (1.6)	
	90–94 years	6	1.6 (0.7)	3	1.6 (0.9)	3	1.6 (0.9)	
	≥95 years	1	0.3 (0.3)	1	0.5 (0.5)	0	0.0 (0.0)	
Comorbidities	HF	54	14.6 (1.8)	27	14.6 (2.6)	27	14.6 (2.6)	0.99
	COPD	255	68.9 (2.4)	128	69.2 (3.4)	127	68.6 (3.4)	
	HF and COPD	61	16.5 (1.9)	30	16.2 (2.7)	31	16.8 (2.7)	
Charlson index	Mean		2.1 (2.4)		2.2 (2.4)		2.0 (2.3)	0.55
	1–2 comorbidities	244	65.9 (2.5)	121	65.4 (3.5)	123	65.5 (3.5)	0.76
	3–4 comorbidities	63	17.0 (2.0)	30	16.2 (2.7)	33	17.8 (2.8)	
	≥5 comorbidities	63	17.0 (2.0)	34	18.4 (2.8)	29	15.7 (2.7)	
Pilot site	Spain	46	12.4 (1.7)	23	12.4 (2.4)	23	12.4 (2.4)	1.00
	England	24	6.5 (1.3)	12	6.5 (1.8)	12	6.5 (1.8)	
	Israel	148	40.0 (2.5)	74	40.0 (3.6)	74	40.0 (3.6)	
	Denmark	152	41.1 (2.6)	76	41.1 (3.6)	76	41.1 (3.6)	
Follow-up months			9.9 (5.3)		7.6 (4.8)		12.3 (4.7)	≤0.01

a Calculated using Fisher’s exact test or chi-square test for categorical variables and Student’s t-test for continuous variables.

**Table 2:** ADLIFE intervention effect presented as hazard ratio and significance.

Resource	ADLIFE intervention effect
Death^a^	1.79 (0.79–4.04)
PC nurse at centre^b^	0.71 (0.47–1.08)
PC nurse by telephone^b^	0.44 (0.26–0.75)**
PC nurse at home^b^	1.04 (0.34–3.17)
PC doctor at centre^b^	0.49 (0.34–0.72)**
PC doctor by telephone^b^	0.60 (0.39–0.93)*
PC doctor at home^b^	0.58 (0.08–3.94)
Outpatient services^b^	1.31 (1.01–1.72)*
Hospitalisation^b^	1.34 (0.84–2.14)
Hospitalisation days^b^	1.60 (0.8–3.19)
Emergency room^c^	0.55 (0.32–0.96)*

a Calculated using logistic regression.

b Calculated using generalized linear model (family:negative binomial, link:log).

c Calculated using hurdle model.

* *p*-value ≤0.05, ** *p*-value ≤0.01.

**Figure 5:**
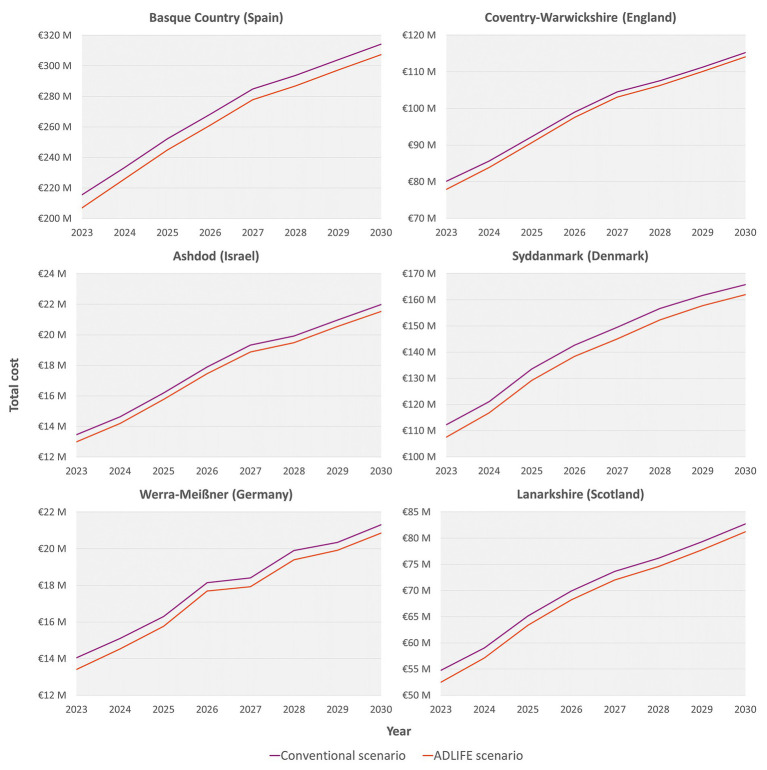
Budget impact analysis (BIA) between conventional and ADLIFE scenarios from 2023 to 2030 at each pilot site.

When medium- to long-term impacts were analysed, as shown in Table 3, projections estimated that disease prevalence in 2030 would increase by 37 to 50%, depending on the pilot site. This will lead to a corresponding increase in resource use and costs, which are detailed in the Supplementary Tables S27–S38. Under the ADLIFE scenario, disease-related costs could be reduced by 1 to 2%, generating cumulative savings between €4 million and €58 million, depending on the specific context of each pilot site, as illustrated in Figure 5. This trend is further reinforced in the scenario analysis, where the ADLIFE intervention reduced the overall burden on ACD patients compared to the conventional scenario, as summarised in Table 4 and expanded in Supplementary Tables S40–S50.

**Table 3:** Evolution of the population with ACD from 2023 to 2030 at each pilot site.

Pilot site	Indicator	Year							
		2023	2024	2025	2026	2027	2028	2029	2030
Basque country (Spain)	Prevalence	42,870	42,560	45,114	47,894	50,743	53,559	56,165	58,920
	Incidence	7,910	10,582	10,708	10,888	11,030	11,186	11,354	11,532
	Mortality	8,220	8,028	7,928	8,039	8,214	8,580	8,599	8,809
Coventry-Warwickshire (England)	Prevalence	14,268	14,861	15,627	16,441	17,320	18,147	18,940	19,768
	Incidence	3,100	3,166	3,222	3,276	3,338	3,404	3,456	3,506
	Mortality	2,507	2,400	2,408	2,397	2,511	2,611	2,628	2,741
Ashdod (Israel)	Prevalence	2,658	2,782	2,943	3,161	3,367	3,584	3,776	3,988
	Incidence	582	602	632	650	668	690	706	728
	Mortality	458	441	414	444	451	498	494	520
Syddanmark (Denmark)	Prevalence	21,092	22,183	23,427	24,699	26,012	27,266	28,562	29,724
	Incidence	4,506	4,590	4,664	4,748	4,842	4,938	5,030	5,118
	Mortality	3,415	3,346	3,392	3,435	3,588	3,642	3,868	3,960
Werra-Meißner (Germany)	Prevalence	2,096	2,156	2,299	2,420	2,547	2,644	2,778	2,888
	Incidence	458	466	470	476	476	480	484	494
	Mortality	398	323	349	349	379	346	374	395
Lanarkshire (Scotland)	Prevalence	9,976	10,617	11,339	12,054	12,772	13,502	14,223	14,944
	Incidence	2,190	2,222	2,256	2,280	2,320	2,352	2,378	2,398
	Mortality	1,549	1,500	1,541	1,562	1,590	1,631	1,657	1,728

**Table 4:** Scenarios analysed in the sensitivity analysis with their description.

Scenario	Description	Cumulative savings
Base case	Intervention effect: estimated	€58 million
Scenario 1	Intervention effect: protective effects halved	€19 million
Scenario 2	Intervention effect: detrimental effects doubled	€54 million
Scenario 3	Intervention effect: protective effects halved and detrimental effects doubled	€14 million
Scenario 4	Scenario 3 # population: 30% higher	€18 million
Scenario 5	Scenario 3 # population: 30% lower	€10 million
Scenario 6	Scenario 3 # resource use: 30% higher	€17 million
Scenario 7	Scenario 3 # resource use: 30% lower	€10 million
Scenario 8	Scenario 3 # unit cost: 30% higher	€18 million
Scenario 8	Scenario 3 # unit cost: 30% lower	€10 million
Scenario 10	Scenario 3 # unit cost: 30% lower for protective effects and 30% higher detrimental effects	€1 million
Scenario 11	Intervention effect: derived from previous works in the literature (C3-CLOUD)	€71 million

## Discussion

This work revealed a promising effect of the ADLIFE intervention on patient care and healthcare resource management. Although it was in place for a relatively short period, the intervention generated meaningful changes in the use of healthcare resources that could have a significant impact on the healthcare system in the future if adopted.

The intervention led to a significant reduction in in-person consultations at the healthcare centre, with a decrease of 51% for PC doctors (Table 2). This suggests that ADLIFE effectively facilitated remote patient monitoring, reducing the need for face-to-face visits (63). Additionally, telephone consultations with both primary care nurses and doctors decreased by 40 and 56%, respectively, highlighting the intervention’s positive impact on communication and follow-up care. In contrast, outpatient visits increased by 31%. These changes may suggest that patients became more aware of their condition, resulting in higher engagement and greater empowerment in managing their health (64), possibly leading them to seek more specialised care or follow-up services. Nonetheless, while improved self-management and communication with healthcare professionals may have encouraged patients to seek specialist attention when necessary, it could also suggest a tendency to pursue specialised advice more frequently, even for less severe concerns, or a shift in workload from primary to specialised care. Such a pattern may have implications for healthcare costs, as increased outpatient consultations could raise expenses. However, if these visits facilitate earlier detection and timely management of complications, they may prevent disease exacerbations and deterioration, reducing the need for emergency room visits or hospital admissions—which are substantially costlier—potentially offsetting expenses in the long term. Regarding emergency room visits at the hospital, the data indicates that ADLIFE had a protective effect. The probability of such visits in the intervention group was half that of the control group, suggesting that the intervention may have helped manage patients’ health more effectively and potentially prevented worsening conditions that lead to emergencies (65, 66). Nevertheless, contrary to findings reported in the literature (66, 67), no statistically significant differences were observed in other healthcare resources, including hospitalisations. The absence of statistically significant differences in hospitalisations and hospitalisation days suggests that the intervention may not have sufficiently influenced the most severe cases. This lack of effect may be attributed to the baseline clinical complexity of the target population, whose health conditions were already advanced, thereby reducing the potential for substantial improvement in this outcome (68, 69). This fact underscores the inherent challenges of managing ACD and indicates a potential area for further research and development (70–72).

The long-term epidemiological results from the pilot sites highlighted a significant challenge ahead (Table 3), with projections indicating that the prevalence of the population suffering from ACD will increase by 37–50% by 2030. This expected rise will inevitably lead to a corresponding increase in the resource utilisation needs of these patients, as well as the associated healthcare costs (73–75). Such trends highlighted the pressing demand for effective healthcare solutions and underlined the necessity for interventions aimed at mitigating these impacts (2, 74, 76). In this context, the BIA showed promising outcomes (Figure 5), where the analysis indicated that the successful adoption of the ADLIFE intervention could lead to a reduction in the overall burden of disease by 1–2%, depending on the pilot site context. This reduction could have been greater if significant differences in hospitalisation had been found, as this is the costliest healthcare resource (66, 67).

Although the findings were encouraging, they should be interpreted with caution, as none of the sites reached the estimated sample size defined in the research protocol. This reduced sample size limits both statistical power and generalisability. A scenario analysis was conducted to test structural assumptions, specifically examining how reducing the beneficial effects and increasing the detrimental effects of the intervention could influence the results (Table 4). The analysis still yielded positive outcomes but stressed the importance of achieving significant improvements in key resource areas, particularly emergency room visits and hospitalisations, as these constituted the costliest resources and emerged as the most critical factors for the long-term sustainability of the healthcare system (65–67). Additionally, a scenario based on effectiveness outcomes reported in the literature and observed in the C3-Cloud project was examined to assess how the impact on the healthcare system might vary in the future under different effectiveness scenarios (22, 62), thus reinforcing the conclusions and robustness of the simulation model. The variability in cumulative savings across different sites was also noteworthy, indicating that such differences were influenced by the characteristics and dimension of the health system under analysis, including differences in population projections and unit costs. This underscored the importance of tailoring interventions to local contexts to maximise their effectiveness (77, 78). Overall, the results from the BIA together with scenario analysis highlighted the potential of the ADLIFE intervention to not only improve resource utilisation but also reduce healthcare costs associated with chronic ACD patients’ management. While these findings should be considered exploratory, they suggested that implementing interventions such as ADLIFE could lead to significant benefits for health systems facing increasing demands from an aging population (74–76).

From another perspective, within the scope of integrated care, the results were also promising, considering that concerns remain regarding care for the ACD population delivered through healthcare systems organised by medical specialties (16, 17). In practice, the specialty leading patient care is often determined by the most complex co-existing condition, but effective management of ACD patients requires diverse expertise (16). However, integrating care across multiple medical specialties and individual providers has proven to be a challenging task (4, 6). Therefore, it is important to acknowledge that, in the context of introducing new technologies such as ADLIFE, the involvement of multiple stakeholders and the need to change the behaviours of the professionals involved made the implementation of the intervention a complex task (79). The success is closely tied to the individuals involved and the organisational structures to which they belong, as various structural, organisational, and professional barriers may foster resistance to change (80, 81). In change management, resistance from both healthcare professionals and the general public has been identified as a key hurdle that can hinder the adoption of new interventions, leading to difficulties, delays, or even implementation failure (82, 83). Increased workload and poor digital literacy are identified as the main barriers at the healthcare provider and patient levels, while efficiency in care delivery and better disease management serve as key facilitators (84). Therefore, if adopted, evidence of ADLIFE’s effectiveness could act as a facilitator at both levels. Furthermore, the Chronic Care Model identifies six areas for improvement in promoting high-quality management of chronic diseases (85)—resources and policies, self-management support, organisation of health care, delivery system design, decision support, clinical information systems—and since the ADLIFE intervention addresses almost all of them, this could further reinforce its influence.

Regarding the method used, DES models were employed to mathematically represent the natural history of the disease (38, 39). DES was particularly well-suited for this purpose, as it explicitly incorporates time and offers the flexibility needed to model both simple and complex interactions, making the approach more generalizable (32, 33). This enabled a dynamic assessment of the long-term progression of the disease burden and the estimation of the ADLIFE intervention’s budgetary impact with reliability and validity (29, 60), providing decision-makers with the necessary information to anticipate and manage the long-term consequences of chronic diseases (29). This also paves the way for the use of continuous improvement tools that support management, such as Deming’s plan-do-study-act (PDSA) cycle (86, 87). Should the ADLIFE intervention be adopted and specific objectives to achieve set, simulation models would make it possible to evaluate any drift in the intervention’s course after implementation (60). Consequently, this technique is especially recommended for evaluating complex interventions where assessing sustainability is a key concern (30). In this sense, the advantages of dynamic modelling for representing complex systems and its application in health services evaluation were repeatedly emphasised and endorsed by various international expert groups (32, 33), who also noted its limited use to date (31, 33).

### Limitations

The main limitation of the study was related to the availability of data. Access to the anonymised database from the Osakidetza-Basque Health Service enabled the development of a robust simulation model, but the unavailability of similar population-level data at other sites limited the creation of context-specific models for each location. Although adapting Basque Country-specific model for application at other sites was a practical and valid approach, it was subject to notable constraints, as it assumed that ACD patients shared similar patterns of disease progression and healthcare use. Consequently, inherent differences between health systems—such as variations in clinical practices, resource availability, and patient interactions with healthcare—were not fully captured by the adapted model. To make models more representative across contexts, future research will need to address key policy challenges related to data access and sharing—both for primary and secondary use—while balancing potential benefits with privacy risks. A commitment to reducing barriers to cross-border data flows, investing in infrastructure and skills, establishing common standards, and fostering trust through stakeholder engagement will be central to achieving this goal (88). The new European Health Data Space (EHDS) regulation may support these efforts in the coming years (89), contributing to a more connected and digital European healthcare landscape (90).

Another limitation was the narrow number of participants recruited, which hindered the ability to obtain conclusive results. However, it is important to consider that the recruitment phase was likely affected by the overburdened healthcare professionals and the inherent challenges of patient enrolment processes (91, 92). Despite the limited sample size, some positive trends and statistically significant differences were observed, suggesting that with a larger sample, more pronounced effects could be detected, potentially even in key outcomes such as hospitalisations (66, 67). Further research should increase sample size and diversify settings to confirm results and strengthen evidence for ACD patients, enabling a better understanding of how digitally supported integrated care positively influences them, and paving the way for the development of guidelines and policy recommendations.

## Conclusion

Long-term epidemiological projections anticipated a significant challenge on the horizon due to a substantial rise in the prevalence of individuals with ACD by 2030, underscoring the need for timely and effective healthcare solutions. In response, the ADLIFE intervention showed promising results in improving patient care and resource management, with economic projections indicating its potential to reduce disease burden and generate sustained savings across different healthcare system contexts. Future work should consider strategies to manage the most severe cases, where ensuring a large sample size and an extended follow-up period, combined with leveraging the EHDS regulation for data access and sharing, could enable the detection of statistically significant and context-specific differences in key resource use, notably hospitalisations.

## Data Availability

The datasets presented in this article are not readily available because given the potentially sensitive nature of the data, the Ethics Committees of the participating healthcare provider organisations did not authorise public access. Dataset access requests should be made through the official channels of each organisation. Requests to access the datasets should be directed to igor.larranagauribeetxebarria@bio-sistemak.eus.

## Group member of ADLIFE consortium

Ana Ortega-Gil, Ángel Moro, Itxaso Alayo, Urko Aguirre, and Bárbara López from Biosistemak, Spain; Dipak Kalra from The European Institute for Innovation Through Health Data (i~HD), Belgium; Oliver Gröne, and Janika Blömeke-Cammin from OptiMedis, Germany; Mikael Lilja from Region Jämtland Härjedalen, Sweden; Tim Robbins University Hospitals Coventry & Warwickshire, United Kingdom; Natassia K. Juul, and Emergency & Medical Department M/FAM from Odense University Hospital, Denmark; Alec Morton, and Konstantinos Koutsouradis from University of Strathclyde, Scotland; Bunyamin Sarigul, and Gokhan Yilmaz from SRDC, Turkey; Omid Pournik, Shramika Panchal, and Ashley Peake from the University of Birmingham, United Kingdom.

## Glossary

ACD: advanced chronic disease
ADLIFE: integrated personalized care for patients with advanced chronic diseases to improve health and quality of life
AIC: Akaike information criterion
BIA: budget impact analysis
COPD: chronic obstructive pulmonary disease
DES: discrete event simulation
EHDS: European Health Data Space
EHR: electronic health records
FAC2: factor of two
FB: fractional bias
FV: fractional variance
GLM: generalized linear model
HC: hospital care
HF: heart failure
HR: hazard ratio
ICD-10: international classification of diseases, tenth revision
ICT: information and communication technologies
NMSE: normalised mean square error
PC: primary care
PDSA: plan-do-study-act
R: correlation coefficient
