# Supplementary material for: Evaluation of the epidemiological and economic impact of the ADLIFE intervention on medium- to long-term in patients with advanced chronic disease
Source: Front Public Health. 2025 Nov 26;13:1682492. doi: 10.3389/fpubh.2025.1682492 (PMC12689975; doi:10.3389/fpubh.2025.1682492)
Supplement: Supplementary file 1 [file Data_Sheet_1.PDF]

Title: Evaluation of the epidemiological and economic impact of the ADLIFE intervention on medium- to long-term in patients with advanced chronic disease. Supplementary material.

Table SM 1 - Descriptive analysis of the target population in the Basque Country, based on data collected from 2012 to 2019.

		N	%
Patients		104,500	
Sex	Women	49,510	47%
	Men	54,990	53%
Age	Mean	76.26 (11.19)	
	55-59 years	11,039	11%
	60-64 years	8,226	8%
	65-69 years	10,040	10%
	70-74 years	12,735	12%
	75-79 years	15,549	15%
	80-84 years	19,102	18%
	85-89 years	17,238	16%
	90-94 years	8,757	8%
	≥95 years	1,814	2%
Comorbidities	HF	78,340	75%
	COPD	27,764	27%
Charlson index	Mean	2.97 (2.13)	
	1-2 comorbidities	55,839	53%
	3-4 comorbidities	30,779	29%
	≥5 comorbidities	17,882	17%

Table SM 2 - Unit costs of different resources obtained from pilot sites and adjusted for the year 2023.

Resource	Spain	England		Israel		Denmark		Germany	Scotland	
	Euro (EUR)	Pound Sterling (GBP)	Euro (EUR)	Israeli Shekel (ILS/NIS)	Euro (EUR)	Danish Krone (DKK)	Euro (EUR)	Euro (EUR)	Pound Sterling (GBP)	Euro (EUR)
PC nurse at centre	13.84	18.16	20.49	56.00	14.92	160.72	21.61	68.28	39.60	44.68
PC nurse by telephone	6.92	12.11	13.66	56.00	14.92	128.14	17.23	68.28	9.56	10.79
PC nurse at home	25.14	24.21	27.32	180.00	47.95	160.72	21.61	68.28	25.30	28.55
PC doctor at centre	31.37	30.27	34.15	75.00	19.98	160.72	21.61	68.28	46.20	52.13
PC doctor by telephone	15.69	18.16	20.49	75.00	19.98	128.14	17.23	68.28	17.38	19.61
PC doctor at home	43.94	60.54	68.30	400.00	106.56	160.72	21.61	68.28	25.30	28.55
Cardiology (first)	153.50	226.41	255.45	126.00	33.57	1,141.00	153.41	95.77	157.31	177.49
Cardiology (successive)	90.27	159.82	180.32	126.00	33.57	1,141.00	153.41	95.77	94.19	106.27
Respiratory (first)	153.50	224.42	253.21	120.00	31.97	1,234.00	165.92	53.41	157.31	177.49
Respiratory (successive)	90.27	170.74	192.64	120.00	31.97	1,234.00	165.92	53.41	94.19	106.27
Endocrinology (first)	181.18	227.62	256.82	131.00	34.90	1,736.00	233.41	55.27	157.31	177.49
Endocrinology (successive)	99.16	170.71	192.61	131.00	34.90	1,736.00	233.41	55.27	103.46	116.73
Nephrology (first)	164.04	212.43	239.69	165.00	43.96	1,576.00	211.90	419.44	157.31	177.49
Nephrology (successive)	93.67	158.69	179.05	165.00	43.96	1,576.00	211.90	419.44	97.73	110.26
Neurology (first)	153.50	224.42	253.21	165.00	43.96	2,559.00	344.07	83.03	157.31	177.49
Neurology (successive)	90.27	170.74	192.64	165.00	43.96	2,559.00	344.07	83.03	94.19	106.27
Psychiatry (first)	139.15	224.42	253.21	117.00	31.17	2,413.00	324.44	96.88	157.31	177.49
Psychiatry (successive)	80.55	170.74	192.64	117.00	31.17	2,413.00	324.44	96.88	84.04	94.83
Internal medicine (first)	155.19	231.25	260.92	75.00	19.98	2,150.00	289.08	69.17	161.92	182.69
Internal medicine (successive)	91.07	193.72	218.57	75.00	19.98	2,150.00	289.08	69.17	95.02	107.21
Emergency room	282.81	170.71	192.61	898.00	239.23	305.00	41.01	135.63	202.53	228.51
Hospitalisation (per day)	599.43	600.00	676.97	2,546.00	678.26	-	-	872.33	570.04	643.17
Hospitalisation (per stay)	6,188.09	-	-	-	-	31,513.00	4,237.10	6,252.06	-	-

Table SM 3 - Population projections for Basque Country region in Spain, as provided by the Basque Statistics Institute (EUSTAT).

		2023	2024	2025	2026	2027	2028	2029	2030
Women	55-59	85,700	85,300	84,600	84,300	84,400	85,000	85,600	86,700
	60-64	81,400	82,200	83,200	83,700	84,400	84,500	84,200	83,500
	65-69	71,600	73,600	76,000	77,800	79,000	79,800	80,700	81,500
	70-74	64,800	64,600	64,800	65,800	67,600	69,400	71,200	73,600
	75-79	57,900	59,400	60,300	60,200	60,300	61,000	61,000	61,300
	80-84	39,600	42,400	45,400	47,600	49,700	51,800	53,200	54,100
	85-89	36,400	34,600	32,500	32,700	31,800	32,100	34,300	37,000
	90-94	22,100	22,800	23,400	23,700	24,200	23,700	22,500	21,300
	≥95	7,800	8,500	9,100	9,900	10,500	11,200	11,900	12,500
Men	55-59	82,800	83,400	83,100	83,300	84,100	84,900	85,600	86,600
	60-64	75,200	76,100	77,200	78,200	79,000	80,200	80,700	80,600
	65-69	64,300	65,900	68,100	69,800	70,800	71,400	72,300	73,500
	70-74	55,500	55,400	55,400	56,200	57,900	59,500	61,000	63,200
	75-79	45,400	46,900	47,900	48,200	48,300	48,900	49,000	49,200
	80-84	27,700	29,600	32,200	33,500	35,300	36,900	38,400	39,300
	85-89	20,300	19,600	18,500	18,900	18,900	19,500	21,000	22,900
	90-94	9,700	10,300	10,700	11,000	11,200	11,100	10,700	10,200
	≥95	2,300	2,600	2,900	3,200	3,700	3,900	4,400	4,600

Table SM 4 - Population projections for Coventry-Warwickshire region in England, as provided by the Office for National Statistics.

		2023	2024	2025	2026	2027	2028	2029	2030
Women	55-59	30,947	31,010	30,835	30,703	30,536	30,164	29,663	28,986
	60-64	27,639	28,443	29,344	29,770	30,073	30,267	30,330	30,164
	65-69	23,314	23,770	24,235	25,227	25,853	26,666	27,448	28,312
	70-74	22,015	21,662	21,498	21,536	21,857	22,176	22,621	23,091
	75-79	21,501	21,542	21,544	21,503	20,708	20,187	19,895	19,767
	80-84	14,301	15,196	15,940	16,574	17,871	18,400	18,488	18,522
	85-89	9,525	9,772	9,907	9,925	10,023	10,522	11,231	11,800
	90-94	4,559	4,594	4,713	4,842	5,010	5,181	5,333	5,433
	≥95	1,495	1,496	1,492	1,527	1,536	1,533	1,541	1,575
Men	55-59	30,131	30,110	29,989	29,914	29,803	29,473	28,972	28,483
	60-64	27,470	28,029	28,482	28,895	29,054	29,019	29,016	28,908
	65-69	22,205	22,737	23,429	24,165	24,970	25,764	26,314	26,764
	70-74	20,206	20,032	19,812	19,870	20,200	20,522	21,030	21,682
	75-79	18,892	19,032	18,977	18,870	18,010	17,702	17,583	17,427
	80-84	11,731	12,320	13,101	13,777	14,836	15,240	15,385	15,390
	85-89	6,821	7,126	7,259	7,285	7,446	7,850	8,303	8,854
	90-94	2,652	2,715	2,825	2,969	3,151	3,308	3,471	3,555
	≥95	504	525	536	555	555	583	591	607

Table SM 5 - Population projections for Ashdod region in Israel, as provided by the Central Bureau of Statistics (CBS).

		2023	2024	2025	2026	2027	2028	2029	2030
Women	55-59	6,058	6,080	6,102	6,280	6,458	6,636	6,814	6,992
	60-64	5,805	5,826	5,847	6,018	6,188	6,359	6,529	6,700
	65-69	5,819	5,840	5,860	5,895	5,930	5,964	5,999	6,034
	70-74	5,329	5,348	5,367	5,398	5,430	5,462	5,494	5,526
	75-79	3,518	3,823	4,128	4,364	4,600	4,835	5,071	5,307
	80-84	2,579	2,803	3,026	3,199	3,372	3,545	3,717	3,890
	85-89	1,764	1,835	1,890	1,940	1,985	2,020	2,061	2,118
	90-94	781	787	808	817	835	862	882	885
	≥95	256	256	256	258	256	255	255	256
Men	55-59	5,644	5,747	5,851	6,032	6,214	6,395	6,577	6,758
	60-64	5,200	5,295	5,390	5,558	5,725	5,892	6,059	6,226
	65-69	4,840	4,897	4,954	5,004	5,054	5,103	5,153	5,202
	70-74	4,264	4,314	4,365	4,409	4,452	4,496	4,539	4,583
	75-79	2,766	3,079	3,392	3,586	3,780	3,974	4,168	4,362
	80-84	1,793	1,996	2,199	2,325	2,450	2,576	2,702	2,828
	85-89	1,217	1,301	1,371	1,416	1,452	1,497	1,533	1,583
	90-94	389	398	421	435	459	470	493	500
	≥95	74	77	80	81	81	83	84	85

Table SM 6 - Population projections for Syddanmark region in Denmark, as provided by the Statistics Denmark.

		2023	2024	2025	2026	2027	2028	2029	2030
Women	55-59	45,489	44,902	43,814	42,749	41,624	41,281	41,112	41,168
	60-64	40,172	41,162	42,151	43,013	44,255	44,724	44,122	43,209
	65-69	38,262	38,254	38,089	38,365	38,491	38,915	39,896	41,062
	70-74	34,560	34,458	34,526	34,877	35,566	36,116	36,134	36,196
	75-79	33,511	34,130	33,864	33,213	32,174	31,377	31,344	31,558
	80-84	21,655	22,647	24,014	25,516	27,012	28,072	28,615	28,462
	85-89	12,588	13,119	13,519	14,092	14,708	15,401	16,199	17,280
	90-94	5,574	5,626	5,780	5,934	6,184	6,575	6,933	7,218
	≥95	1,828	1,832	1,829	1,872	1,896	1,946	2,004	2,092
Men	55-59	46,033	45,501	44,398	43,187	42,079	41,466	41,181	40,839
	60-64	39,996	40,952	42,044	43,138	44,471	44,910	44,357	43,333
	65-69	36,858	36,953	37,192	37,319	37,526	38,218	39,165	40,371
	70-74	32,768	32,677	32,703	33,082	33,658	33,882	34,062	34,439
	75-79	30,314	30,840	30,593	29,897	28,971	28,524	28,568	28,743
	80-84	17,827	18,650	19,857	21,461	22,896	23,747	24,246	24,150
	85-89	8,543	9,106	9,517	9,954	10,445	11,139	11,772	12,648
	90-94	2,731	2,786	2,922	3,055	3,299	3,499	3,783	3,995
	≥95	519	539	554	571	581	617	644	682

Table SM 7 - Population projections for Werra-Meißner region in Germany, as provided by the Federal Statistical Office (DESTATIS).

		2023	2024	2025	2026	2027	2028	2029	2030
Women	55-59	4,266	4,196	4,083	3,942	3,769	3,577	3,409	3,280
	60-64	4,416	4,530	4,616	4,670	4,689	4,659	4,582	4,459
	65-69	3,753	3,859	3,985	4,110	4,236	4,373	4,489	4,578
	70-74	3,312	3,312	3,318	3,366	3,430	3,523	3,623	3,743
	75-79	2,470	2,553	2,760	2,879	2,930	2,951	2,953	2,964
	80-84	2,493	2,368	2,127	2,004	2,038	2,084	2,163	2,341
	85-89	1,674	1,778	1,860	1,884	1,820	1,759	1,678	1,511
	90-94	606	634	680	731	784	847	907	954
	≥95	199	197	203	203	199	199	214	233
Men	55-59	4,193	4,108	3,976	3,813	3,620	3,397	3,217	3,083
	60-64	4,280	4,402	4,490	4,553	4,569	4,543	4,451	4,308
	65-69	3,568	3,693	3,832	3,961	4,092	4,241	4,372	4,461
	70-74	2,927	2,921	2,935	3,004	3,090	3,203	3,320	3,453
	75-79	2,056	2,121	2,286	2,364	2,392	2,389	2,390	2,408
	80-84	1,872	1,786	1,610	1,517	1,539	1,581	1,644	1,771
	85-89	1,068	1,144	1,203	1,228	1,190	1,155	1,112	1,000
	90-94	293	316	346	381	419	453	491	518
	≥95	63	65	71	69	71	74	80	92

Table SM 8 - Population projections for Lanarkshire region in Scotland, as provided by the National Records of Scotland.

		2023	2024	2025	2026	2027	2028	2029	2030
Women	55-59	26,558	26,330	26,004	25,873	25,527	24,661	23,662	22,796
	60-64	24,664	25,212	25,665	25,851	25,947	25,968	25,753	25,433
	65-69	21,062	21,637	22,132	22,583	23,096	23,669	24,213	24,659
	70-74	17,473	17,611	17,960	18,471	18,984	19,501	20,057	20,542
	75-79	14,605	14,980	15,400	15,603	15,289	15,234	15,399	15,743
	80-84	9,965	10,133	10,153	10,364	11,131	11,559	11,875	12,212
	85-89	6,125	6,222	6,277	6,220	6,258	6,423	6,550	6,567
	90-94	2,109	2,101	2,113	2,123	2,147	2,170	2,185	2,183
	≥95	691	684	669	670	658	642	632	633
Men	55-59	24,425	24,354	23,963	23,813	23,314	22,650	21,795	21,099
	60-64	22,597	23,020	23,388	23,385	23,457	23,480	23,424	23,063
	65-69	18,685	19,009	19,665	20,205	20,772	21,197	21,612	21,980
	70-74	15,313	15,490	15,552	15,966	16,409	16,754	17,077	17,702
	75-79	12,210	12,542	12,913	13,064	12,709	12,727	12,921	13,007
	80-84	7,134	7,408	7,569	7,872	8,612	9,026	9,296	9,573
	85-89	3,707	3,831	3,933	3,938	4,043	4,174	4,369	4,482
	90-94	1,167	1,187	1,223	1,262	1,300	1,350	1,390	1,415
	≥95	222	230	232	236	229	238	237	242

Table SM 9 - Prevalent and incident cohorts of ACD population in the Basque Country region of Spain from 2012 to 2019, obtained from Basque Health Service databases.

		Prevalence	Incidence							
		2012	2012	2013	2014	2015	2016	2017	2018	2019
Women	55-59	1,145	156	190	215	215	344	397	424	501
	60-64	917	112	117	131	139	301	339	374	428
	65-69	1,325	130	151	163	182	323	396	398	423
	70-74	2,092	233	220	242	233	458	461	473	486
	75-79	3,092	466	420	383	453	555	530	507	614
	80-84	3,331	804	719	746	818	933	991	871	820
	85-89	2,399	863	792	863	909	1,048	1,154	1,103	1,160
	90-94	861	480	485	482	589	705	786	803	800
	≥95	96	106	118	129	119	150	192	211	220
Men	55-59	2,938	361	330	432	425	711	689	728	838
	60-64	1,889	226	276	259	285	572	656	631	574
	65-69	2,109	309	299	333	370	883	824	718	704
	70-74	2,478	347	311	358	442	1,039	1,026	950	886
	75-79	2,816	554	485	470	519	974	908	880	923
	80-84	2,169	654	634	688	722	1,256	1,118	938	890
	85-89	1,110	501	454	596	587	976	981	881	861
	90-94	275	192	210	235	269	386	407	371	421
	≥95	25	41	42	39	38	66	76	71	75

Table SM 10 - Parameters of the regression models used to set patients input characteristics.

		HF	COPD	Charlson group		Pharmacy cost
				1-2	3-4	
Sex	Women	0.000	0.000	0.000	0.000	0.000
	Men	-0.771	1.138	-0.713	-0.340	0.007
Age group	55-59	0.000	0.000	0.000	0.000	0.000
	60-64	0.027	-0.274	-0.261	-0.010	0.296
	65-69	0.274	-0.083	-0.494	-0.051	0.554
	70-74	0.624	-0.180	-0.663	-0.082	0.648
	75-79	1.153	-0.421	-0.795	-0.087	0.790
	80-84	1.566	-0.215	-0.967	-0.113	0.763
	85-89	1.892	-0.023	-1.084	-0.079	0.690
	90-94	2.189	-0.061	-1.094	0.030	0.470
	≥95	2.438	0.024	-1.118	0.033	0.180
HF	No	0.000	0.000	0.000	0.000	0.000
	Yes	0.000	-25.069	-1.792	-0.568	0.599
COPD	No	0.000	0.000	0.000	0.000	0.000
	Yes	0.000	0.000	-1.622	-0.448	0.428
Charlson group	1-2	0.000	0.000	0.000	0.000	0.000
	3-4	0.000	0.000	0.000	0.000	0.438
	≥5	0.000	0.000	0.000	0.000	0.623
Constant		0.608	20.676	4.050	1.357	5.866

Table SM 11 - Distributions and parameters of the time-to-event functions for primary care.

		PC nurse						PC doctor					
		Centre		Telephone		Home		Centre		Telephone		Home	
		First	Between	First	Between	First	Between	First	Between	First	Between	First	Between
Type of function		Weibull	Weibull	Weibull	Weibull	Weibull	Weibull	Weibull	Weibull	Weibull	Weibull	Weibull	Weibull
Sex	Women	0.000	0.000	0.000	0.000	0.000	0.000	0.000	0.000	0.000	0.000	0.000	0.000
	Men	0.119	0.071	-0.055	-0.014	-0.173	-0.009	0.049	-0.007	-0.163	-0.113	-0.185	0.037
Age group	55-59	0.000	0.000	0.000	0.000	0.000	0.000	0.000	0.000	0.000	0.000	0.000	0.000
	60-64	0.238	0.067	0.205	0.066	0.284	0.104	0.184	0.049	0.070	0.012	0.336	-0.003
	65-69	0.316	0.102	0.260	0.107	0.538	0.163	0.140	0.104	0.058	0.105	0.596	0.098
	70-74	0.402	0.145	0.349	0.177	0.812	0.221	0.179	0.150	0.140	0.182	0.967	0.155
	75-79	0.408	0.138	0.421	0.213	1.056	0.244	0.170	0.158	0.215	0.263	1.220	0.193
	80-84	0.356	0.133	0.543	0.267	1.340	0.306	0.141	0.147	0.362	0.367	1.513	0.268
	85-89	0.248	0.066	0.701	0.310	1.649	0.321	0.087	0.092	0.525	0.466	1.815	0.352
	90-94	0.074	0.096	0.799	0.359	1.880	0.379	-0.051	0.063	0.626	0.547	2.097	0.465
	≥95	-0.080	0.086	0.883	0.540	2.087	0.503	-0.184	0.162	0.783	0.734	2.366	0.631
HF	No	0.000	0.000	0.000	0.000	0.000	0.000	0.000	0.000	0.000	0.000	0.000	0.000
	Yes	0.330	0.330	0.398	0.121	0.383	0.131	0.123	0.116	0.128	0.036	0.282	0.100
COPD	No	0.000	0.000	0.000	0.000	0.000	0.000	0.000	0.000	0.000	0.000	0.000	0.000
	Yes	0.139	0.066	0.426	0.093	-0.097	0.005	0.245	0.039	0.280	0.070	-0.085	0.075
Charlson group	1-2	0.000	0.000	0.000	0.000	0.000	0.000	0.000	0.000	0.000	0.000	0.000	0.000
	3-4	0.116	0.032	0.211	0.079	0.153	0.074	0.106	0.047	0.202	0.096	0.132	0.025
	≥5	0.178	0.085	0.423	0.198	0.353	0.169	0.124	0.076	0.370	0.188	0.289	0.044
Constant		-4.208	-3.293	-5.303	-2.904	-5.537	-2.417	-2.664	-3.104	-4.228	-2.883	-7.061	-2.796
Beta		0.524	0.743	0.549	0.586	0.552	0.582	0.538	0.798	0.625	0.572	0.652	0.606

Table SM 12 - Distributions and parameters of the time-to-event functions for outpatient services.

		Outpatient services													
		Cardiology		Respiratory		Endocrinology		Nephrology		Neurology		Psychiatry		Internal medicine	
		First	Between	First	Between	First	Between	First	Between	First	Between	First	Between	First	Between
Type of function		Weibull	Weibull	Weibull	Weibull	Weibull	Weibull	Weibull	Weibull	Weibull	Weibull	Weibull	Weibull	Weibull	Weibull
Sex	Women	0.000	0.000	0.000	0.000	0.000	0.000	0.000	0.000	0.000	0.000	0.000	0.000	0.000	0.000
	Men	0.298	0.071	0.195	-0.021	-0.447	0.090	0.212	0.051	-0.058	0.021	-0.523	0.084	0.106	0.002
Age group	55-59	0.000	0.000	0.000	0.000	0.000	0.000	0.000	0.000	0.000	0.000	0.000	0.000	0.000	0.000
	60-64	0.173	-0.028	0.209	-0.018	-0.052	0.004	0.000	0.029	0.167	0.002	-0.288	-0.170	0.177	0.012
	65-69	0.205	-0.073	0.196	-0.015	-0.161	0.055	0.019	-0.110	0.316	0.005	-0.608	-0.161	0.322	0.046
	70-74	0.198	-0.094	0.162	-0.012	-0.350	0.045	-0.051	-0.110	0.485	0.043	-0.746	-0.224	0.363	0.045
	75-79	0.112	-0.111	0.065	-0.009	-0.603	0.039	-0.208	-0.198	0.602	0.027	-0.891	-0.251	0.449	0.035
	80-84	-0.001	-0.083	-0.145	-0.017	-0.982	0.115	-0.494	-0.285	0.598	0.050	-1.109	-0.252	0.448	0.057
	85-89	-0.340	-0.059	-0.554	0.010	-1.554	0.168	-0.965	-0.317	0.378	0.102	-1.382	-0.202	0.303	0.078
	90-94	-0.923	0.006	-1.117	0.069	-2.331	0.133	-1.568	-0.365	-0.183	0.129	-1.646	-0.091	-0.002	0.089
	≥95	-1.503	0.211	-1.578	0.135	-3.133	0.653	-2.665	-0.309	-0.613	0.231	-1.669	0.104	-0.572	0.232
HF	No	0.000	0.000	0.000	0.000	0.000	0.000	0.000	0.000	0.000	0.000	0.000	0.000	0.000	0.000
	Yes	1.031	0.223	0.290	0.094	0.193	0.049	0.493	0.144	-0.101	-0.021	-0.003	0.102	0.658	0.130
COPD	No	0.000	0.000	0.000	0.000	0.000	0.000	0.000	0.000	0.000	0.000	0.000	0.000	0.000	0.000
	Yes	-0.135	0.135	1.373	0.184	-0.116	0.042	-0.453	0.053	-0.076	0.133	0.313	0.287	0.255	0.031
Charlson group	1-2	0.000	0.000	0.000	0.000	0.000	0.000	0.000	0.000	0.000	0.000	0.000	0.000	0.000	0.000
	3-4	0.119	0.086	0.051	0.017	0.603	0.029	1.119	0.313	0.253	0.024	0.227	0.089	0.230	-0.002
	≥5	0.135	0.130	-0.018	0.068	1.232	0.167	1.866	0.457	0.394	0.040	0.382	0.046	0.402	0.049
Constant		-5.327	-5.343	-5.922	-5.409	-5.896	-5.886	-7.321	-4.294	-7.020	-5.791	-5.697	-3.371	-7.412	-4.135
Beta		0.506	0.989	0.507	1.045	0.509	1.124	0.484	0.871	0.642	1.079	0.470	0.832	0.523	0.912

Table SM 13 - Distributions and parameters of the time-to-event functions for emergency room, hospitalisation and death.

		Emergency room		Hospitalisation		Death
		First	Between	First	Between	
Type of function		Weibull	Weibull	Weibull	Weibull	Gompertz
Sex	Women	0.000	0.000	0.000	0.000	0.000
	Men	-0.002	0.037	0.119	0.042	0.216
Age group	55-59	0.000	0.000	0.000	0.000	0.000
	60-64	0.098	0.018	0.169	-0.003	0.306
	65-69	0.171	0.041	0.312	-0.016	0.531
	70-74	0.258	0.068	0.432	0.001	0.819
	75-79	0.461	0.081	0.551	-0.006	1.075
	80-84	0.504	0.096	0.645	-0.003	1.408
	85-89	0.684	0.123	0.775	0.032	1.824
	90-94	0.631	0.180	0.865	0.097	2.319
	≥95	0.638	0.376	1.006	0.313	3.173
HF	No	0.000	0.000	0.000	0.000	0.000
	Yes	0.299	0.006	0.699	0.033	0.618
COPD	No	0.000	0.000	0.000	0.000	0.000
	Yes	0.170	0.085	0.238	0.130	0.042
Charlson group	1-2	0.000	0.000	0.000	0.000	0.000
	3-4	0.196	0.096	0.180	0.088	0.174
	≥5	0.389	0.186	0.362	0.160	0.389
Constant		-5.739	-3.656	-6.684	-4.156	-9.891
Beta		0.711	0.700	0.666	0.746	0.000

Table SM 14 - Goodness-of-fit test of the simulation model under the conventional scenario for the years 2012 to 2014.

2012															
Metric	Nurse Centre	Nurse Telf.	Nurse Home	Doctor Centre	Doctor Telf.	Doctor Home	Car.	Res.	End.	Nep.	Neu.	Psy.	Int. med.	Emerg. room	Hosp.	Death
**R: > 0.8**	0.867	0.879	0.873	0.880	0.884	0.878	0.847	0.879	0.841	0.832	0.839	0.865	0.840	0.876	0.887	0.885
**NMSE: < 0.5**	0.041	0.031	0.023	0.008	0.128	0.136	0.065	0.016	0.132	0.054	0.093	0.067	0.065	0.028	0.002	0.009
**FB: [-0.5, 0.5]**	0.137	-0.133	0.017	0.039	-0.321	0.265	0.150	0.059	0.234	-0.023	0.157	0.119	0.134	0.132	-0.003	0.074
**FV: [-0.5, 0.5]**	0.258	0.193	0.190	0.150	0.241	0.311	0.345	0.180	0.448	0.359	0.377	0.315	0.336	0.182	0.078	0.090
**FAC2: > 0.8**	1.000	1.000	1.000	1.000	1.000	1.000	1.000	1.000	0.917	1.000	0.917	1.000	1.000	1.000	1.000	1.000
2013																
Metric	Nurse Centre	Nurse Telf.	Nurse Home	Doctor Centre	Doctor Telf.	Doctor Home	Car.	Res.	End.	Nep.	Neu.	Psy.	Int. med.	Emerg. room	Hosp.	Death
**R: > 0.8**	0.873	0.879	0.884	0.879	0.882	0.886	0.841	0.873	0.857	0.855	0.852	0.870	0.873	0.886	0.885	0.884
**NMSE: < 0.5**	0.013	0.017	0.010	0.008	0.035	0.006	0.040	0.015	0.037	0.033	0.039	0.041	0.020	0.002	0.008	0.006
**FB: [-0.5, 0.5]**	0.029	-0.085	0.042	-0.031	-0.162	0.017	0.057	-0.049	0.058	-0.021	-0.016	-0.108	0.078	-0.011	-0.059	-0.016
**FV: [-0.5, 0.5]**	0.192	0.161	0.120	0.153	0.150	0.092	0.330	0.189	0.292	0.281	0.291	0.217	0.188	0.083	0.122	0.117
**FAC2: > 0.8**	1.000	1.000	1.000	1.000	1.000	1.000	1.000	1.000	1.000	1.000	1.000	0.833	0.917	1.000	1.000	1.000
**2014**																
Metric	Nurse Centre	Nurse Telf.	Nurse Home	Doctor Centre	Doctor Telf.	Doctor Home	Car.	Res.	End.	Nep.	Neu.	Psy.	Int. med.	Emerg. room	Hosp.	Death
**R: > 0.8**	0.878	0.887	0.888	0.883	0.886	0.886	0.850	0.885	0.864	0.840	0.867	0.879	0.878	0.885	0.882	0.879
**NMSE: < 0.5**	0.029	0.023	0.002	0.007	0.006	0.006	0.030	0.010	0.025	0.045	0.032	0.081	0.010	0.009	0.013	0.019
**FB: [-0.5, 0.5]**	-0.124	-0.133	0.012	-0.045	0.041	-0.035	-0.024	-0.082	-0.001	0.052	-0.101	-0.232	0.006	-0.061	-0.087	0.093
**FV: [-0.5, 0.5]**	0.203	0.118	0.061	0.126	0.114	0.086	0.299	0.099	0.250	0.332	0.229	0.204	0.159	0.134	0.137	0.153
**FAC2: > 0.8**	1.000	1.000	1.000	1.000	1.000	1.000	1.000	1.000	1.000	1.000	1.000	0.833	0.917	1.000	1.000	1.000

Table SM 15 - Goodness-of-fit test of the simulation model under the conventional scenario for the years 2015 to 2017.

2015																
Metric	Nurse Centre	Nurse Telf.	Nurse Home	Doctor Centre	Doctor Telf.	Doctor Home	Car.	Res.	End.	Nep.	Neu.	Psy.	Int. med.	Emerg. room	Hosp.	Death
**R: > 0.8**	0.884	0.886	0.883	0.884	0.887	0.886	0.855	0.883	0.859	0.814	0.881	0.873	0.881	0.886	0.878	0.888
**NMSE: < 0.5**	0.016	0.002	0.011	0.006	0.024	0.004	0.027	0.013	0.028	0.066	0.043	0.081	0.026	0.004	0.009	0.001
**FB: [-0.5, 0.5]**	-0.090	0.005	0.022	-0.046	0.118	-0.023	-0.043	-0.088	-0.021	-0.005	-0.151	-0.199	-0.082	-0.043	-0.037	-0.001
**FV: [-0.5, 0.5]**	0.162	0.083	0.138	0.119	0.170	0.081	0.280	0.123	0.258	0.412	0.226	0.231	0.224	0.095	0.156	0.049
**FAC2: > 0.8**	1.000	1.000	1.000	1.000	1.000	1.000	0.917	1.000	1.000	1.000	1.000	0.917	1.000	1.000	1.000	1.000
2016																
Metric	Nurse Centre	Nurse Telf.	Nurse Home	Doctor Centre	Doctor Telf.	Doctor Home	Car.	Res.	End.	Nep.	Neu.	Psy.	Int. med.	Emerg. room	Hosp.	Death
**R: > 0.8**	0.888	0.885	0.878	0.887	0.887	0.883	0.871	0.885	0.883	0.866	0.884	0.882	0.884	0.879	0.872	0.887
**NMSE: < 0.5**	0.005	0.009	0.016	0.001	0.013	0.011	0.034	0.005	0.007	0.021	0.021	0.021	0.019	0.006	0.010	0.028
**FB: [-0.5, 0.5]**	-0.058	0.059	-0.031	-0.003	0.069	-0.049	-0.125	0.023	-0.033	-0.018	-0.120	-0.074	-0.081	-0.019	-0.007	-0.101
**FV: [-0.5, 0.5]**	0.072	0.130	0.169	0.069	0.171	0.120	0.243	0.105	0.115	0.233	0.135	0.141	0.187	0.151	0.197	0.201
**FAC2: > 0.8**	1.000	1.000	1.000	1.000	1.000	1.000	1.000	0.917	1.000	1.000	1.000	1.000	1.000	1.000	1.000	1.000
2017																
Metric	Nurse Centre	Nurse Telf.	Nurse Home	Doctor Centre	Doctor Telf.	Doctor Home	Car.	Res.	End.	Nep.	Neu.	Psy.	Int. med.	Emerg. room	Hosp.	Death
**R: > 0.8**	0.886	0.883	0.864	0.888	0.887	0.878	0.881	0.887	0.884	0.882	0.882	0.882	0.874	0.877	0.861	0.887
**NMSE: < 0.5**	0.002	0.018	0.040	0.003	0.013	0.018	0.006	0.007	0.007	0.007	0.019	0.019	0.011	0.006	0.015	0.039
**FB: [-0.5, 0.5]**	-0.017	0.075	-0.010	0.037	0.069	-0.042	-0.027	0.060	-0.032	0.038	-0.087	-0.052	0.004	0.004	-0.012	-0.156
**FV: [-0.5, 0.5]**	0.079	0.198	0.270	0.072	0.171	0.168	0.135	0.100	0.116	0.125	0.176	0.144	0.184	0.164	0.251	0.185
**FAC2: > 0.8**	1.000	1.000	1.000	1.000	1.000	1.000	1.000	1.000	1.000	0.917	1.000	1.000	1.000	1.000	1.000	1.000

Table SM 16 - Goodness-of-fit test of the simulation model under the conventional scenario for the years 2018 to 2019.

2018																
Metric	Nurse Centre	Nurse Telf.	Nurse Home	Doctor Centre	Doctor Telf.	Doctor Home	Car.	Res.	End.	Nep.	Neu.	Psy.	Int. med.	Emerg. room	Hosp.	Death
R: > 0.8	0.883	0.880	0.855	0.888	0.884	0.871	0.884	0.887	0.883	0.883	0.869	0.886	0.861	0.878	0.853	0.888
NMSE: < 0.5	0.004	0.009	0.051	0.009	0.019	0.026	0.003	0.002	0.006	0.008	0.017	0.009	0.022	0.008	0.018	0.020
FB: [-0.5, 0.5]	0.024	0.021	-0.011	0.076	0.090	-0.017	-0.014	-0.006	0.007	0.044	-0.024	0.034	0.050	0.052	0.019	-0.119
FV: [-0.5, 0.5]	0.122	0.171	0.320	0.123	0.196	0.221	0.103	0.069	0.117	0.121	0.214	0.096	0.258	0.162	0.285	0.120
FAC2: > 0.8	1.000	1.000	1.000	1.000	1.000	1.000	1.000	1.000	1.000	1.000	1.000	1.000	1.000	1.000	1.000	1.000
2019																
Metric	Nurse Centre	Nurse Telf.	Nurse Home	Doctor Centre	Doctor Telf.	Doctor Home	Car.	Res.	End.	Nep.	Neu.	Psy.	Int. med.	Emerg. room	Hosp.	Death
R: > 0.8	0.887	0.874	0.846	0.887	0.881	0.863	0.885	0.887	0.887	0.887	0.881	0.883	0.856	0.872	0.836	0.885
NMSE: < 0.5	0.008	0.010	0.056	0.005	0.011	0.038	0.005	0.003	0.010	0.012	0.008	0.061	0.028	0.010	0.025	0.052
FB: [-0.5, 0.5]	-0.075	-0.020	-0.007	0.054	0.049	-0.070	-0.033	-0.024	0.081	0.096	0.039	0.133	0.074	0.046	0.020	-0.207
FV: [-0.5, 0.5]	0.086	0.192	0.348	0.103	0.190	0.258	0.115	0.073	0.085	0.084	0.135	0.206	0.274	0.199	0.347	0.151
FAC2: > 0.8	1.000	1.000	1.000	1.000	1.000	1.000	1.000	1.000	1.000	1.000	1.000	1.000	1.000	1.000	1.000	1.000

Table SM 17 - Trends in the prevalence and incidence of ACD population in the Basque Country region of Spain from 2023 to 2030.

		Prevalence	Incidence							
		2023	2023	2024	2025	2026	2027	2028	2029	2030
Women	55-59	955	753	749	743	741	742	747	752	762
	60-64	1,793	524	529	536	539	543	544	542	538
	65-69	2,059	544	559	577	591	600	606	613	619
	70-74	2,240	552	550	552	561	576	591	607	627
	75-79	2,534	629	645	655	654	655	663	663	666
	80-84	2,799	537	575	616	645	674	702	721	734
	85-89	3,938	564	536	504	507	493	497	531	573
	90-94	3,516	351	362	372	376	384	376	357	338
	≥95	1,329	99	108	116	126	133	142	151	159
Men	55-59	1,678	467	470	469	470	474	479	483	488
	60-64	2,606	428	433	439	445	450	456	459	459
	65-69	2,990	393	403	416	427	433	436	442	449
	70-74	3,279	396	395	395	401	413	425	435	451
	75-79	3,437	502	519	530	533	534	541	542	544
	80-84	2,892	592	633	688	716	754	789	821	840
	85-89	2,706	814	786	742	758	758	782	842	918
	90-94	1,696	760	807	838	862	878	870	838	799
	≥95	415	295	333	372	410	475	500	564	590

Table SM 18 - Trends in the prevalence and incidence of ACD population in the Coventry-Warwickshire region of England from 2023 to 2030.

		Prevalence	Incidence							
		2023	2023	2024	2025	2026	2027	2028	2029	2030
Women	55-59	345	272	272	271	270	268	265	261	255
	60-64	609	178	183	189	192	193	195	195	194
	65-69	670	177	181	184	192	196	203	208	215
	70-74	761	188	184	183	184	186	189	193	197
	75-79	941	234	234	234	234	225	219	216	215
	80-84	1,011	194	206	216	225	242	249	251	251
	85-89	1,030	148	151	154	154	155	163	174	183
	90-94	725	72	73	75	77	79	82	85	86
	≥95	255	19	19	19	19	19	19	20	20
Men	55-59	611	170	170	169	169	168	166	163	161
	60-64	952	156	159	162	164	165	165	165	165
	65-69	1,033	136	139	143	148	153	157	161	163
	70-74	1,194	144	143	141	142	144	147	150	155
	75-79	1,430	209	211	210	209	199	196	194	193
	80-84	1,225	251	263	280	294	317	326	329	329
	85-89	909	273	286	291	292	299	315	333	355
	90-94	464	208	213	221	233	247	259	272	278
	≥95	91	65	67	69	71	71	75	76	78

Table SM 19 - Trends in the prevalence and incidence of ACD population in the Ashdod region of Israel from 2023 to 2030.

		Prevalence	Incidence							
		2023	2023	2024	2025	2026	2027	2028	2029	2030
Women	55-59	68	53	53	54	55	57	58	60	61
	60-64	128	37	37	38	39	40	41	42	43
	65-69	167	44	44	44	45	45	45	46	46
	70-74	184	45	46	46	46	46	47	47	47
	75-79	154	38	42	45	47	50	53	55	58
	80-84	182	35	38	41	43	46	48	50	53
	85-89	191	27	28	29	30	31	31	32	33
	90-94	124	12	12	13	13	13	14	14	14
	≥95	44	3	3	3	3	3	3	3	3
Men	55-59	114	32	32	33	34	35	36	37	38
	60-64	180	30	30	31	32	33	34	34	35
	65-69	225	30	30	30	31	31	31	32	32
	70-74	252	30	31	31	31	32	32	32	33
	75-79	209	31	34	38	40	42	44	46	48
	80-84	187	38	43	47	50	52	55	58	60
	85-89	162	49	52	55	57	58	60	61	63
	90-94	68	30	31	33	34	36	37	39	39
	≥95	13	9	10	10	10	10	11	11	11

Table SM 20 - Trends in the prevalence and incidence of ACD population in the Syddanmark region of Denmark from 2023 to 2030.

		Prevalence	Incidence							
		2023	2023	2024	2025	2026	2027	2028	2029	2030
Women	55-59	507	400	394	385	376	366	363	361	362
	60-64	885	259	265	272	277	285	288	284	278
	65-69	1,100	291	291	289	291	292	296	303	312
	70-74	1,195	294	293	294	297	303	308	308	308
	75-79	1,467	364	371	368	361	349	341	341	343
	80-84	1,531	294	307	326	346	366	380	388	386
	85-89	1,362	195	203	210	218	228	238	251	268
	90-94	887	89	89	92	94	98	104	110	115
	≥95	311	23	23	23	24	24	25	25	27
Men	55-59	933	260	256	251	244	237	234	232	230
	60-64	1,386	228	233	239	245	253	255	252	247
	65-69	1,714	225	226	227	228	230	233	239	247
	70-74	1,936	234	233	233	236	240	242	243	246
	75-79	2,295	335	341	339	331	320	316	316	318
	80-84	1,861	381	399	424	459	489	508	518	516
	85-89	1,139	343	365	382	399	419	447	472	507
	90-94	478	214	218	229	239	259	274	296	313
	≥95	94	67	69	71	73	75	79	83	87

Table SM 21 - Trends in the prevalence and incidence of ACD population in the Werra-Meißner region of Germany from 2023 to 2030.

		Prevalence	Incidence							
		2023	2023	2024	2025	2026	2027	2028	2029	2030
Women	55-59	48	37	37	36	35	33	31	30	29
	60-64	97	28	29	30	30	30	30	29	29
	65-69	108	29	29	30	31	32	33	34	35
	70-74	114	28	28	28	29	29	30	31	32
	75-79	108	27	28	30	31	32	32	32	32
	80-84	176	34	32	29	27	28	28	29	32
	85-89	181	26	28	29	29	28	27	26	23
	90-94	96	10	10	11	12	12	13	14	15
	≥95	34	3	3	3	3	3	3	3	3
Men	55-59	85	24	23	22	22	20	19	18	17
	60-64	148	24	25	26	26	26	26	25	25
	65-69	166	22	23	23	24	25	26	27	27
	70-74	173	21	21	21	21	22	23	24	25
	75-79	156	23	23	25	26	26	26	26	27
	80-84	195	40	38	34	32	33	34	35	38
	85-89	142	43	46	48	49	48	46	45	40
	90-94	51	23	25	27	30	33	35	38	41
	≥95	11	8	8	9	9	9	10	10	12

Table SM 22 - Trends in the prevalence and incidence of ACD population in the Lanarkshire region of Scotland from 2023 to 2030.

		Prevalence	Incidence							
		2023	2023	2024	2025	2026	2027	2028	2029	2030
Women	55-59	296	233	231	228	227	224	217	208	200
	60-64	543	159	162	165	166	167	167	166	164
	65-69	606	160	164	168	172	175	180	184	187
	70-74	604	149	150	153	157	162	166	171	175
	75-79	639	159	163	167	170	166	166	167	171
	80-84	704	135	137	138	140	151	157	161	166
	85-89	663	95	96	97	96	97	99	101	102
	90-94	336	33	33	34	34	34	34	35	35
	≥95	118	9	9	9	9	8	8	8	8
Men	55-59	495	138	137	135	134	131	128	123	119
	60-64	783	129	131	133	133	134	134	133	131
	65-69	869	114	116	120	124	127	129	132	134
	70-74	905	109	110	111	114	117	120	122	126
	75-79	924	135	139	143	144	141	141	143	144
	80-84	745	152	158	162	168	184	193	199	205
	85-89	494	149	154	158	158	162	167	175	180
	90-94	204	91	93	96	99	102	106	109	111
	≥95	40	28	29	30	30	29	31	30	31

Table SM 23 - Mortality, polypharmacy and the number of patients with at least one contact with healthcare resources for intervention and control groups during the follow-up period.

Resource	Intervention (N=185)		Control (N=185)		p-value[a]
	N	%	N	%	
Death	14	7.6%	15	8.1%	1.000
Polypharmacy*	149	80.5%	145	78.4%	0.699
Contacts with PC nurse	70	37.8%	86	46.5%	0.114
At centre	50	27.0%	75	40.5%	0.008
At home	33	17.8%	55	29.7%	0.010
By telephone	16	8.6%	15	8.1%	1.000
Contacts with PC doctor	85	45.9%	91	49.2%	0.603
At centre	77	41.6%	87	47.0%	0.346
At home	58	31.4%	73	39.5%	0.128
By telephone	6	3.2%	4	2.2%	0.751
Contacts with outpatient services	95	51.4%	135	73.0%	0.000
Cardiology	22	11.9%	47	25.4%	0.001
Respiratory	69	37.3%	101	54.6%	0.001
Endocrinology	0	0.0%	17	9.2%	0.000
Nephrology	7	3.8%	10	5.4%	0.621
Neurology	8	4.3%	12	6.5%	0.491
Psychiatry	1	0.5%	6	3.2%	0.121
Internal medicine	6	3.2%	1	0.5%	0.121
Contacts with emergency room	46	24.9%	67	36.2%	0.024
Hospitalisation	36	19.5%	51	27.6%	0.086

[a] Calculated using Fisher's exact test.

* Defined as the prescription of five or more chronic medications.

Table SM 24 - Healthcare resource use per patient for intervention and control groups during the follow-up period.

Resource	Intervention (N=185)		Control (N=185)		p-value[a]
	Mean	SD	Mean	SD	
Contacts with PC nurse	1.41	2.75	3.32	6.86	0.001
At centre	0.70	1.54	1.89	4.71	0.001
By telephone	0.31	0.83	0.94	2.44	0.001
At home	0.39	1.77	0.49	2.26	0.664
Contacts with PC doctor	3.05	4.55	9.18	12.42	0.000
At centre	1.71	2.81	5.83	8.60	0.000
By telephone	1.30	2.64	3.30	6.17	0.000
At home	0.05	0.30	0.05	0.43	0.888
Contacts with outpatient services	1.21	1.84	1.98	2.53	0.001
Cardiology	0.24	1.12	0.64	1.36	0.002
Respiratory	0.69	1.09	0.85	1.09	0.153
Endocrinology	0.00	0.00	0.22	0.83	0.000
Nephrology	0.05	0.31	0.12	0.64	0.215
Neurology	0.05	0.24	0.09	0.37	0.240
Psychiatry	0.01	0.07	0.05	0.36	0.071
Internal medicine	0.17	1.07	0.01	0.15	0.043
Contacts with emergency room	0.46	0.99	0.70	1.39	0.059
Hospitalisation	0.33	0.81	0.55	1.21	0.044
Hospitalisation days	1.92	6.69	2.74	7.54	0.272

[a] Calculated using Student's t-test.

Table SM 25 - Effect of the ADLIFE intervention on the probability of death and the probability of contact with different healthcare resources.

Resource	β	SE	EXP(β)	p-value[a]
Death	0.580	0.417	1.786	0.164
Polypharmacy*	0.888	0.296	2.430	0.270
PC nurse	-0.066	0.270	0.936	0.808
At centre	-0.294	0.267	0.746	0.272
By telephone	-0.536	0.305	0.585	0.079
At home	0.670	0.427	1.955	0.116
PC doctor	0.355	0.269	1.426	0.186
At centre	0.224	0.264	1.251	0.396
By telephone	-0.007	0.263	0.993	0.979
At home	0.901	0.741	2.461	0.224
Outpatient services	-0.406	0.239	0.666	0.089
Hospitalisation	0.026	0.266	1.027	0.921

[a] p-value calculated using logistic regression.

* Defined as the prescription of five or more chronic medications.

Table SM 26 - Effect of the ADLIFE intervention on the number of contacts with different healthcare resources.

Resource	β	SE	EXP(β)	p-value[a]
PC nurse	-0.417	0.191	0.659	0.030
At centre	-0.339	0.212	0.713	0.110
By telephone	-0.814	0.272	0.443	0.003
At home	0.040	0.569	1.041	0.944
PC doctor	-0.613	0.187	0.542	0.001
At centre	-0.703	0.191	0.495	0.000
By telephone	-0.502	0.219	0.605	0.022
At home	-0.551	0.981	0.576	0.574
Outpatient services	0.274	0.136	1.315	0.045
Hospitalisation	0.294	0.239	1.341	0.220
Hospitalisation days	0.468	0.353	1.596	0.185

[a] p-value calculated using generalized linear model (family:negative binomial, link:log).

Table SM 27 - Evolution of the number of contacts with different healthcare resources from 2023 to 2030 at the Spain pilot site.

		2023	2024	2025	2026	2027	2028	2029	2030
Conventional scenario	PC doctor	496,106	541,790	564,947	586,121	601,958	611,997	619,456	631,011
	PC doctor at centre	356,209	386,947	401,772	411,144	418,151	423,474	425,927	431,772
	PC doctor by telephone	100,320	103,751	107,335	118,046	125,913	130,920	136,253	142,968
	PC doctor at home	39,577	51,092	55,840	56,931	57,894	57,603	57,276	56,271
	PC nurse	451,321	469,723	494,264	519,152	535,547	552,096	567,998	585,269
	PC doctor at centre	228,322	243,279	254,209	262,937	271,012	280,225	289,020	300,503
	PC doctor by telephone	56,191	64,429	71,968	78,926	84,254	88,579	93,388	98,292
	PC doctor at home	166,808	162,015	168,087	177,289	180,281	183,292	185,590	186,474
	Outpatient services	73,291	91,640	104,913	114,507	122,554	133,765	142,677	149,982
	Cardiology	24,880	29,220	33,898	37,169	39,387	41,354	43,316	45,211
	Respiratory	13,281	17,402	20,782	23,466	25,292	30,322	34,521	37,752
	Endocrinology	5,470	6,910	8,017	9,084	10,005	10,696	11,102	11,229
	Nephrology	7,218	8,601	8,723	8,653	9,176	9,664	9,766	10,447
	Neurology	5,457	7,504	8,718	9,546	10,098	11,315	12,075	12,761
	Psychiatry	6,017	8,809	11,162	13,070	14,949	16,310	17,405	18,379
	Internal medicine	10,968	13,194	13,613	13,519	13,647	14,104	14,492	14,203
	Emergency room	48,712	56,298	61,177	64,789	67,677	69,146	71,204	72,399
	Hospitalisation	21,938	24,155	26,920	29,221	31,795	32,654	33,940	35,159
ADLIFE scenario	PC doctor	355,698	434,919	392,786	500,322	518,575	532,582	543,236	552,332
	PC doctor at centre	247,451	307,335	336,946	352,278	362,471	369,965	376,572	380,579
	PC doctor by telephone	68,670	76,492	81,350	91,113	98,210	105,014	109,388	115,482
	PC doctor at home	39,577	51,092	55,840	56,931	57,894	57,603	57,276	56,271
	PC nurse	422,429	437,429	459,469	481,729	495,711	510,389	524,426	540,486
	PC doctor at centre	228,322	243,279	254,209	262,937	271,012	280,225	289,020	300,503
	PC doctor by telephone	27,299	32,135	37,173	41,503	44,418	46,872	49,816	53,509
	PC doctor at home	166,808	162,015	168,087	177,289	180,281	183,292	185,590	186,474
	Outpatient services	83,391	103,146	117,435	127,869	137,202	149,446	159,681	167,958
	Cardiology	31,278	36,112	41,149	45,009	47,697	50,002	52,241	54,381
	Respiratory	16,983	22,016	26,053	28,988	31,630	37,355	42,600	46,558
	Endocrinology	5,470	6,910	8,017	9,084	10,005	10,696	11,102	11,229
	Nephrology	7,218	8,601	8,723	8,653	9,176	9,664	9,766	10,447
	Neurology	5,457	7,504	8,718	9,546	10,098	11,315	12,075	12,761
	Psychiatry	6,017	8,809	11,162	13,070	14,949	16,310	17,405	18,379
	Internal medicine	10,968	13,194	13,613	13,519	13,647	14,104	14,492	14,203
	Emergency room	29,229	36,810	40,704	44,174	46,830	48,108	49,981	50,647
	Hospitalisation	21,938	24,155	26,920	29,221	31,795	32,654	33,940	35,159

Table SM 28 - Evolution of the number of contacts with different healthcare resources from 2023 to 2030 at the England pilot site.

		2023	2024	2025	2026	2027	2028	2029	2030
Conventional scenario	PC doctor	169,269	183,985	190,739	195,361	200,548	204,314	205,447	208,150
	PC doctor at centre	124,015	134,611	137,235	138,021	141,116	142,348	142,669	143,176
	PC doctor by telephone	32,566	33,457	35,234	38,622	40,661	42,814	44,335	46,405
	PC doctor at home	12,688	15,917	18,270	18,718	18,771	19,152	18,443	18,569
	PC nurse	151,567	158,797	166,665	173,178	178,170	184,465	189,789	194,783
	PC doctor at centre	80,186	85,768	88,442	90,885	92,989	95,906	98,385	100,936
	PC doctor by telephone	18,676	21,443	23,734	25,825	26,860	28,766	30,968	32,082
	PC doctor at home	52,705	51,586	54,489	56,468	58,321	59,793	60,436	61,765
	Outpatient services	26,285	32,125	36,868	40,067	42,882	47,325	49,926	51,690
	Cardiology	8,758	10,223	11,745	12,545	13,750	14,273	14,889	15,214
	Respiratory	4,832	6,340	7,396	8,386	8,846	10,921	12,440	13,101
	Endocrinology	2,065	2,529	2,738	3,209	3,583	3,812	3,795	4,187
	Nephrology	2,815	3,017	3,245	3,155	3,180	3,498	3,612	3,627
	Neurology	1,963	2,648	3,047	3,364	3,506	4,028	4,347	4,422
	Psychiatry	2,159	2,950	3,816	4,613	5,184	5,625	5,759	6,034
	Internal medicine	3,693	4,418	4,881	4,795	4,833	5,168	5,084	5,105
	Emergency room	16,659	18,817	20,818	21,981	23,085	23,237	23,898	24,325
	Hospitalisation	7,441	8,019	8,861	9,837	10,566	10,782	11,232	11,718
ADLIFE scenario	PC doctor	121,108	147,939	134,709	168,398	173,539	178,625	182,164	183,618
	PC doctor at centre	86,277	107,299	116,439	119,742	122,673	125,650	127,746	127,652
	PC doctor by telephone	22,143	24,723	26,163	29,938	32,095	33,823	35,975	37,397
	PC doctor at home	12,688	15,917	18,270	18,718	18,771	19,152	18,443	18,569
	PC nurse	141,974	148,186	155,325	160,918	165,638	171,068	174,846	179,516
	PC doctor at centre	80,186	85,768	88,442	90,885	92,989	95,906	98,385	100,936
	PC doctor by telephone	9,083	10,832	12,394	13,565	14,328	15,369	16,025	16,815
	PC doctor at home	52,705	51,586	54,489	56,468	58,321	59,793	60,436	61,765
	Outpatient services	30,019	36,158	41,079	44,906	48,208	52,570	55,641	57,700
	Cardiology	11,088	12,668	14,116	15,421	16,712	17,093	17,873	18,185
	Respiratory	6,236	7,928	9,236	10,349	11,210	13,346	15,171	16,140
	Endocrinology	2,065	2,529	2,738	3,209	3,583	3,812	3,795	4,187
	Nephrology	2,815	3,017	3,245	3,155	3,180	3,498	3,612	3,627
	Neurology	1,963	2,648	3,047	3,364	3,506	4,028	4,347	4,422
	Psychiatry	2,159	2,950	3,816	4,613	5,184	5,625	5,759	6,034
	Internal medicine	3,693	4,418	4,881	4,795	4,833	5,168	5,084	5,105
	Emergency room	10,111	12,362	13,915	15,019	15,852	16,241	16,861	17,383
	Hospitalisation	7,441	8,019	8,861	9,837	10,566	10,782	11,232	11,718

Table SM 29 - Evolution of the number of contacts with different healthcare resources from 2023 to 2030 at the Israel pilot site.

		2023	2024	2025	2026	2027	2028	2029	2030
Conventional scenario	PC doctor	31,094	34,359	36,173	37,611	39,171	40,334	41,317	42,146
	PC doctor at centre	23,020	25,264	26,582	26,780	27,581	28,412	28,764	29,278
	PC doctor by telephone	5,858	6,069	6,397	7,427	8,099	8,476	9,025	9,293
	PC doctor at home	2,216	3,026	3,194	3,404	3,491	3,446	3,528	3,575
	PC nurse	28,465	29,325	31,439	32,819	34,826	35,519	37,754	38,794
	PC doctor at centre	15,124	16,072	16,951	17,152	18,399	18,501	19,770	20,520
	PC doctor by telephone	3,701	4,199	4,619	4,805	5,253	5,492	5,908	6,429
	PC doctor at home	9,640	9,054	9,869	10,862	11,174	11,526	12,076	11,845
	Outpatient services	4,767	6,165	7,111	7,872	8,681	9,410	9,893	10,548
	Cardiology	1,577	1,945	2,123	2,445	2,756	2,913	3,001	3,123
	Respiratory	931	1,260	1,500	1,767	1,856	2,222	2,448	2,738
	Endocrinology	397	489	577	634	685	738	766	774
	Nephrology	390	498	597	570	628	636	616	744
	Neurology	332	502	609	656	686	798	887	927
	Psychiatry	373	510	700	838	1,095	1,091	1,156	1,260
	Internal medicine	767	961	1,005	962	975	1,012	1,019	982
	Emergency room	3,030	3,462	3,853	4,223	4,380	4,580	4,834	4,814
	Hospitalisation	1,258	1,409	1,639	1,881	2,089	2,130	2,265	2,403
ADLIFE scenario	PC doctor	22,444	27,624	25,530	32,338	33,911	35,385	36,901	37,291
	PC doctor at centre	16,266	20,118	22,336	23,250	24,104	25,202	26,049	26,076
	PC doctor by telephone	3,962	4,480	4,800	5,684	6,316	6,737	7,324	7,640
	PC doctor at home	2,216	3,026	3,194	3,404	3,491	3,446	3,528	3,575
	PC nurse	26,509	27,192	29,139	30,594	32,352	32,997	34,921	35,797
	PC doctor at centre	15,124	16,072	16,951	17,152	18,399	18,501	19,770	20,520
	PC doctor by telephone	1,745	2,066	2,319	2,580	2,779	2,970	3,075	3,432
	PC doctor at home	9,640	9,054	9,869	10,862	11,174	11,526	12,076	11,845
	Outpatient services	5,482	6,904	8,031	8,933	9,617	10,462	11,011	11,663
	Cardiology	2,002	2,381	2,635	3,095	3,309	3,466	3,558	3,682
	Respiratory	1,221	1,563	1,908	2,178	2,239	2,721	3,009	3,294
	Endocrinology	397	489	577	634	685	738	766	774
	Nephrology	390	498	597	570	628	636	616	744
	Neurology	332	502	609	656	686	798	887	927
	Psychiatry	373	510	700	838	1,095	1,091	1,156	1,260
	Internal medicine	767	961	1,005	962	975	1,012	1,019	982
	Emergency room	1,797	2,209	2,586	2,889	2,932	3,211	3,442	3,400
	Hospitalisation	1,258	1,409	1,639	1,881	2,089	2,130	2,265	2,403

Table SM 30 - Evolution of the number of contacts with different healthcare resources from 2023 to 2030 at the Denmark pilot site.

		2023	2024	2025	2026	2027	2028	2029	2030
Conventional scenario	PC doctor	248,004	271,100	283,021	291,549	298,104	304,210	308,487	311,029
	PC doctor at centre	184,665	199,448	207,124	209,649	211,879	213,501	215,173	214,645
	PC doctor by telephone	46,339	49,213	51,313	55,625	59,458	63,655	66,074	68,914
	PC doctor at home	17,000	22,439	24,584	26,275	26,767	27,054	27,240	27,470
	PC nurse	220,233	231,850	246,497	257,181	267,160	277,187	285,339	293,162
	PC doctor at centre	119,399	126,712	132,793	135,971	140,011	144,606	148,720	153,173
	PC doctor by telephone	26,805	30,535	34,973	38,427	41,298	44,152	46,154	48,798
	PC doctor at home	74,029	74,603	78,731	82,783	85,851	88,429	90,465	91,191
	Outpatient services	39,948	49,332	56,809	61,255	65,511	71,225	75,518	78,030
	Cardiology	13,374	15,639	17,949	19,744	20,664	21,742	22,488	22,969
	Respiratory	7,581	9,411	11,349	12,550	13,811	16,292	18,459	20,015
	Endocrinology	3,151	3,839	4,393	5,007	5,442	5,831	6,080	6,026
	Nephrology	4,015	4,681	4,879	4,616	4,873	5,269	5,285	5,471
	Neurology	2,897	4,093	4,793	5,173	5,365	6,072	6,618	6,853
	Psychiatry	3,194	4,551	6,038	6,991	7,936	8,350	8,944	9,176
	Internal medicine	5,736	7,118	7,408	7,174	7,420	7,669	7,644	7,520
	Emergency room	24,114	28,061	30,580	32,962	34,443	35,447	35,568	36,546
	Hospitalisation	10,776	11,548	13,418	14,695	15,532	16,439	16,962	17,398
ADLIFE scenario	PC doctor	177,376	217,778	199,544	250,648	259,460	266,247	273,250	275,215
	PC doctor at centre	128,596	159,924	174,960	181,367	186,181	188,997	192,711	191,746
	PC doctor by telephone	31,780	35,415	38,815	43,006	46,512	50,196	53,299	55,999
	PC doctor at home	17,000	22,439	24,584	26,275	26,767	27,054	27,240	27,470
	PC nurse	206,489	216,779	229,301	238,600	247,383	256,179	263,561	270,262
	PC doctor at centre	119,399	126,712	132,793	135,971	140,011	144,606	148,720	153,173
	PC doctor by telephone	13,061	15,464	17,777	19,846	21,521	23,144	24,376	25,898
	PC doctor at home	74,029	74,603	78,731	82,783	85,851	88,429	90,465	91,191
	Outpatient services	45,364	55,667	63,520	68,608	73,102	79,404	84,210	86,694
	Cardiology	16,804	19,350	21,826	23,807	25,163	26,134	27,021	27,381
	Respiratory	9,567	12,035	14,183	15,840	16,903	20,079	22,618	24,267
	Endocrinology	3,151	3,839	4,393	5,007	5,442	5,831	6,080	6,026
	Nephrology	4,015	4,681	4,879	4,616	4,873	5,269	5,285	5,471
	Neurology	2,897	4,093	4,793	5,173	5,365	6,072	6,618	6,853
	Psychiatry	3,194	4,551	6,038	6,991	7,936	8,350	8,944	9,176
	Internal medicine	5,736	7,118	7,408	7,174	7,420	7,669	7,644	7,520
	Emergency room	14,558	18,307	20,327	22,517	23,395	24,403	25,456	26,415
	Hospitalisation	10,776	11,548	13,418	14,695	15,532	16,439	16,962	17,398

Table SM 31 - Evolution of the number of contacts with different healthcare resources from 2023 to 2030 at the Germany pilot site.

		2023	2024	2025	2026	2027	2028	2029	2030
Conventional scenario	PC doctor	24,371	26,947	27,987	28,826	29,413	30,142	30,223	30,299
	PC doctor at centre	17,938	19,835	20,144	20,528	20,607	20,911	20,936	20,875
	PC doctor by telephone	4,581	4,791	5,394	5,670	6,196	6,546	6,640	6,797
	PC doctor at home	1,852	2,321	2,449	2,628	2,610	2,685	2,647	2,627
	PC nurse	22,317	23,561	25,095	25,613	26,293	27,564	28,300	28,570
	PC doctor at centre	11,733	12,631	13,236	13,527	13,733	14,257	14,638	14,917
	PC doctor by telephone	2,900	3,312	3,627	3,661	4,062	4,394	4,528	4,704
	PC doctor at home	7,684	7,618	8,232	8,425	8,498	8,913	9,134	8,949
	Outpatient services	3,560	4,695	5,423	5,828	6,184	6,874	7,378	7,594
	Cardiology	1,195	1,450	1,646	1,844	1,927	2,085	2,277	2,255
	Respiratory	697	921	1,087	1,251	1,329	1,531	1,725	1,849
	Endocrinology	317	380	510	532	542	563	582	603
	Nephrology	275	384	422	385	416	479	520	546
	Neurology	270	424	465	465	518	562	591	625
	Psychiatry	242	400	497	650	686	874	818	891
	Internal medicine	564	736	796	701	766	780	865	825
	Emergency room	2,320	2,742	2,906	3,138	3,513	3,674	3,502	3,503
	Hospitalisation	1,002	1,094	1,217	1,463	1,456	1,642	1,670	1,793
ADLIFE scenario	PC doctor	17,718	21,662	19,299	25,177	25,659	26,279	27,229	26,849
	PC doctor at centre	12,711	15,806	16,850	18,058	18,223	18,378	18,919	18,863
	PC doctor by telephone	3,155	3,535	3,876	4,491	4,826	5,216	5,663	5,359
	PC doctor at home	1,852	2,321	2,449	2,628	2,610	2,685	2,647	2,627
	PC nurse	20,821	21,885	23,254	23,878	24,481	25,552	26,141	26,361
	PC doctor at centre	11,733	12,631	13,236	13,527	13,733	14,257	14,638	14,917
	PC doctor by telephone	1,404	1,636	1,786	1,926	2,250	2,382	2,369	2,495
	PC doctor at home	7,684	7,618	8,232	8,425	8,498	8,913	9,134	8,949
	Outpatient services	4,084	5,312	6,182	6,430	6,975	7,610	8,216	8,366
	Cardiology	1,504	1,814	2,087	2,221	2,372	2,540	2,699	2,665
	Respiratory	912	1,174	1,405	1,476	1,675	1,812	2,141	2,211
	Endocrinology	317	380	510	532	542	563	582	603
	Nephrology	275	384	422	385	416	479	520	546
	Neurology	270	424	465	465	518	562	591	625
	Psychiatry	242	400	497	650	686	874	818	891
	Internal medicine	564	736	796	701	766	780	865	825
	Emergency room	1,421	1,705	1,916	2,143	2,381	2,543	2,475	2,594
	Hospitalisation	1,002	1,094	1,217	1,463	1,456	1,642	1,670	1,793

Table SM 32 - Evolution of the number of contacts with different healthcare resources from 2023 to 2030 at the Scotland pilot site.

		2023	2024	2025	2026	2027	2028	2029	2030
Conventional scenario	PC doctor	117,616	128,948	134,085	140,166	143,388	147,140	150,333	153,460
	PC doctor at centre	88,075	96,010	98,672	101,884	102,963	104,903	105,595	107,304
	PC doctor by telephone	21,742	22,814	24,201	26,527	28,311	29,780	32,293	33,475
	PC doctor at home	7,799	10,124	11,212	11,755	12,114	12,457	12,445	12,681
	PC nurse	102,234	109,772	116,017	121,560	127,205	132,673	137,763	142,021
	PC doctor at centre	56,603	60,856	63,491	65,461	68,060	70,779	73,370	75,378
	PC doctor by telephone	12,348	14,533	16,475	18,157	19,505	20,628	22,013	23,360
	PC doctor at home	33,283	34,383	36,051	37,942	39,640	41,266	42,380	43,283
	Outpatient services	19,025	23,773	27,650	29,881	31,797	34,888	38,182	39,820
	Cardiology	6,353	7,307	8,626	9,277	10,056	10,427	11,267	11,688
	Respiratory	3,620	4,733	5,640	6,337	6,671	8,148	9,445	10,172
	Endocrinology	1,575	1,927	2,328	2,551	2,838	3,008	3,093	3,187
	Nephrology	2,062	2,371	2,482	2,463	2,410	2,545	2,682	2,681
	Neurology	1,336	1,939	2,298	2,474	2,602	2,932	3,267	3,344
	Psychiatry	1,567	2,230	2,922	3,577	3,871	4,313	4,616	4,812
	Internal medicine	2,512	3,266	3,354	3,202	3,349	3,515	3,812	3,936
	Emergency room	11,480	12,994	14,315	15,473	16,280	17,040	17,644	17,904
	Hospitalisation	4,953	5,391	6,308	6,988	7,476	7,668	8,024	8,474
ADLIFE scenario	PC doctor	84,488	103,696	94,951	119,993	124,599	128,785	132,364	135,925
	PC doctor at centre	62,065	76,659	83,739	88,020	90,382	92,688	94,141	96,086
	PC doctor by telephone	14,624	16,913	18,118	20,218	22,103	23,640	25,778	27,158
	PC doctor at home	7,799	10,124	11,212	11,755	12,114	12,457	12,445	12,681
	PC nurse	95,746	102,391	107,940	113,015	117,982	123,306	127,757	131,475
	PC doctor at centre	56,603	60,856	63,491	65,461	68,060	70,779	73,370	75,378
	PC doctor by telephone	5,860	7,152	8,398	9,612	10,282	11,261	12,007	12,814
	PC doctor at home	33,283	34,383	36,051	37,942	39,640	41,266	42,380	43,283
	Outpatient services	21,707	26,786	30,940	33,400	35,749	39,042	42,518	44,447
	Cardiology	8,023	9,141	10,496	11,270	12,180	12,686	13,561	13,959
	Respiratory	4,632	5,912	7,060	7,863	8,499	10,043	11,487	12,528
	Endocrinology	1,575	1,927	2,328	2,551	2,838	3,008	3,093	3,187
	Nephrology	2,062	2,371	2,482	2,463	2,410	2,545	2,682	2,681
	Neurology	1,336	1,939	2,298	2,474	2,602	2,932	3,267	3,344
	Psychiatry	1,567	2,230	2,922	3,577	3,871	4,313	4,616	4,812
	Internal medicine	2,512	3,266	3,354	3,202	3,349	3,515	3,812	3,936
	Emergency room	6,971	8,539	9,348	10,522	11,126	11,852	12,421	12,817
	Hospitalisation	4,953	5,391	6,308	6,988	7,476	7,668	8,024	8,474

Table SM 33 - Budget impact analysis from 2023 to 2030 at the Spain pilot site.

		2023	2024	2025	2026	2027	2028	2029	2030
Conventional scenario	Primary care	€22.2 M	€23.9 M	€25.0 M	€25.9 M	€26.5 M	€27.0 M	€27.3 M	€27.8 M
	PC nurse	€7.7 M	€7.9 M	€8.2 M	€8.6 M	€8.9 M	€9.1 M	€9.3 M	€9.5 M
	PC doctor	€14.5 M	€16.0 M	€16.7 M	€17.3 M	€17.6 M	€17.9 M	€18.0 M	€18.3 M
	Hospital care	€116.3 M	€129.8 M	€144.4 M	€156.3 M	€169.1 M	€174.3 M	€181.3 M	€187.6 M
	Outpatient services	€6.6 M	€8.3 M	€9.5 M	€10.3 M	€11.0 M	€12.1 M	€12.9 M	€13.5 M
	Emergency room	€13.8 M	€15.9 M	€17.3 M	€18.3 M	€19.1 M	€19.6 M	€20.1 M	€20.5 M
	Hospitalisation	€95.9 M	€105.5 M	€117.6 M	€127.7 M	€138.9 M	€142.7 M	€148.3 M	€153.6 M
	Drug prescription	€75.6 M	€78.0 M	€80.6 M	€83.8 M	€86.9 M	€90.1 M	€93.0 M	€96.4 M
	Total	€214.1 M	€231.7 M	€250.0 M	€266.0 M	€282.5 M	€291.3 M	€301.6 M	€311.8 M
ADLIFE scenario	Primary care	€18.1 M	€20.7 M	€22.3 M	€23.4 M	€24.0 M	€24.6 M	€25.1 M	€25.4 M
	PC nurse	€7.5 M	€7.7 M	€8.0 M	€8.4 M	€8.6 M	€8.8 M	€9.0 M	€9.2 M
	PC doctor	€10.6 M	€13.1 M	€14.3 M	€15.0 M	€15.5 M	€15.8 M	€16.0 M	€16.2 M
	Hospital care	€111.7 M	€125.3 M	€139.7 M	€151.7 M	€164.5 M	€169.8 M	€176.8 M	€183.1 M
	Outpatient services	€7.6 M	€9.3 M	€10.6 M	€11.5 M	€12.4 M	€13.5 M	€14.4 M	€15.1 M
	Emergency room	€8.3 M	€10.4 M	€11.5 M	€12.5 M	€13.2 M	€13.6 M	€14.1 M	€14.3 M
	Hospitalisation	€95.9 M	€105.5 M	€117.6 M	€127.7 M	€138.9 M	€142.7 M	€148.3 M	€153.6 M
	Drug prescription	€75.6 M	€78.0 M	€80.6 M	€83.8 M	€86.9 M	€90.1 M	€93.0 M	€96.4 M
	Total	€205.4 M	€224.0 M	€242.7 M	€258.8 M	€275.4 M	€284.4 M	€294.9 M	€304.9 M
Difference		-€8.7 M	-€7.6 M	-€7.3 M	-€7.2 M	-€7.0 M	-€6.9 M	-€6.7 M	-€6.9 M

Table SM 34 - Budget impact analysis from 2023 to 2030 at the England pilot site.

		2023	2024	2025	2026	2027	2028	2029	2030
Conventional scenario	Primary care	€9.1 M	€9.8 M	€10.3 M	€10.5 M	€10.8 M	€11.0 M	€11.1 M	€11.3 M
	PC nurse	€3.3 M	€3.5 M	€3.6 M	€3.8 M	€3.9 M	€4.0 M	€4.1 M	€4.2 M
	PC doctor	€5.8 M	€6.4 M	€6.7 M	€6.8 M	€6.9 M	€7.0 M	€7.0 M	€7.1 M
	Hospital care	€44.9 M	€49.3 M	€54.8 M	€60.4 M	€64.8 M	€66.7 M	€69.5 M	€72.4 M
	Outpatient services	€5.0 M	€6.1 M	€7.0 M	€7.6 M	€8.2 M	€9.0 M	€9.5 M	€9.9 M
	Emergency room	€3.2 M	€3.6 M	€4.0 M	€4.2 M	€4.4 M	€4.5 M	€4.6 M	€4.7 M
	Hospitalisation	€36.7 M	€39.6 M	€43.7 M	€48.5 M	€52.1 M	€53.2 M	€55.4 M	€57.8 M
	Drug prescription	€26.1 M	€26.5 M	€27.2 M	€28.0 M	€28.9 M	€29.8 M	€30.6 M	€31.6 M
	Total	€80.1 M	€85.7 M	€92.3 M	€99.0 M	€104.5 M	€107.6 M	€111.3 M	€115.2 M
ADLIFE scenario	Primary care	€7.5 M	€8.6 M	€9.2 M	€9.6 M	€9.8 M	€10.1 M	€10.2 M	€10.4 M
	PC nurse	€3.2 M	€3.3 M	€3.5 M	€3.6 M	€3.7 M	€3.8 M	€3.9 M	€4.0 M
	PC doctor	€4.3 M	€5.3 M	€5.8 M	€6.0 M	€6.1 M	€6.3 M	€6.4 M	€6.4 M
	Hospital care	€44.4 M	€48.8 M	€54.2 M	€60.0 M	€64.4 M	€66.3 M	€69.3 M	€72.1 M
	Outpatient services	€5.7 M	€6.9 M	€7.8 M	€8.5 M	€9.2 M	€10.0 M	€10.6 M	€11.0 M
	Emergency room	€1.9 M	€2.4 M	€2.7 M	€2.9 M	€3.1 M	€3.1 M	€3.2 M	€3.3 M
	Hospitalisation	€36.7 M	€39.6 M	€43.7 M	€48.5 M	€52.1 M	€53.2 M	€55.4 M	€57.8 M
	Drug prescription	€26.1 M	€26.5 M	€27.2 M	€28.0 M	€28.9 M	€29.8 M	€30.6 M	€31.6 M
	Total	€77.9 M	€83.9 M	€90.7 M	€97.6 M	€103.1 M	€106.2 M	€110.1 M	€114.1 M
Difference		-€2.2 M	-€1.8 M	-€1.6 M	-€1.4 M	-€1.4 M	-€1.3 M	-€1.2 M	-€1.1 M

Table SM 35 - Budget impact analysis from 2023 to 2030 at the Israel pilot site.

		2023	2024	2025	2026	2027	2028	2029	2030
Conventional scenario	Primary care	€1.6 M	€1.7 M	€1.8 M	€1.9 M	€2.0 M	€2.0 M	€2.1 M	€2.1 M
	PC nurse	€0.7 M	€0.7 M	€0.8 M	€0.8 M	€0.9 M	€0.9 M	€1.0 M	€1.0 M
	PC doctor	€0.8 M	€0.9 M	€1.0 M	€1.0 M	€1.1 M	€1.1 M	€1.1 M	€1.2 M
	Hospital care	€7.1 M	€8.0 M	€9.3 M	€10.6 M	€11.7 M	€11.9 M	€12.7 M	€13.4 M
	Outpatient services	€0.2 M	€0.2 M	€0.2 M	€0.3 M	€0.3 M	€0.3 M	€0.3 M	€0.4 M
	Emergency room	€0.7 M	€0.8 M	€0.9 M	€1.0 M	€1.0 M	€1.1 M	€1.2 M	€1.2 M
	Hospitalisation	€6.2 M	€7.0 M	€8.1 M	€9.3 M	€10.3 M	€10.5 M	€11.2 M	€11.9 M
	Drug prescription	€4.8 M	€4.9 M	€5.1 M	€5.4 M	€5.6 M	€5.9 M	€6.1 M	€6.4 M
	Total	€13.4 M	€14.6 M	€16.1 M	€17.8 M	€19.3 M	€19.8 M	€20.9 M	€21.9 M
ADLIFE scenario	Primary care	€1.4 M	€1.5 M	€1.6 M	€1.8 M	€1.8 M	€1.9 M	€2.0 M	€2.0 M
	PC nurse	€0.7 M	€0.7 M	€0.8 M	€0.8 M	€0.9 M	€0.9 M	€0.9 M	€0.9 M
	PC doctor	€0.6 M	€0.8 M	€0.9 M	€0.9 M	€1.0 M	€1.0 M	€1.0 M	€1.1 M
	Hospital care	€6.8 M	€7.7 M	€9.0 M	€10.3 M	€11.3 M	€11.6 M	€12.4 M	€13.1 M
	Outpatient services	€0.2 M	€0.2 M	€0.3 M	€0.3 M	€0.3 M	€0.3 M	€0.4 M	€0.4 M
	Emergency room	€0.4 M	€0.5 M	€0.6 M	€0.7 M	€0.7 M	€0.8 M	€0.8 M	€0.8 M
	Hospitalisation	€6.2 M	€7.0 M	€8.1 M	€9.3 M	€10.3 M	€10.5 M	€11.2 M	€11.9 M
	Drug prescription	€4.8 M	€4.9 M	€5.1 M	€5.4 M	€5.6 M	€5.9 M	€6.1 M	€6.4 M
	Total	€12.9 M	€14.1 M	€15.7 M	€17.4 M	€18.8 M	€19.4 M	€20.5 M	€21.5 M
Difference		-€0.5 M	-€0.4 M	-€0.4 M	-€0.4 M	-€0.5 M	-€0.4 M	-€0.4 M	-€0.4 M

Table SM 36 - Budget impact analysis from 2023 to 2030 at the Denmark pilot site.

		2023	2024	2025	2026	2027	2028	2029	2030
Conventional scenario	Primary care	€9.8 M	€10.5 M	€11.1 M	€11.4 M	€11.8 M	€12.1 M	€12.3 M	€12.5 M
	PC nurse	€4.6 M	€4.9 M	€5.2 M	€5.4 M	€5.6 M	€5.8 M	€6.0 M	€6.1 M
	PC doctor	€5.2 M	€5.6 M	€5.9 M	€6.1 M	€6.2 M	€6.3 M	€6.4 M	€6.4 M
	Hospital care	€64.1 M	€71.2 M	€81.8 M	€89.2 M	€94.3 M	€99.7 M	€102.9 M	€105.6 M
	Outpatient services	€8.6 M	€10.8 M	€12.4 M	€13.4 M	€14.3 M	€15.5 M	€16.4 M	€16.9 M
	Emergency room	€9.9 M	€11.5 M	€12.5 M	€13.5 M	€14.1 M	€14.5 M	€14.6 M	€15.0 M
	Hospitalisation	€45.7 M	€48.9 M	€56.9 M	€62.3 M	€65.8 M	€69.7 M	€71.9 M	€73.7 M
	Drug prescription	€38.4 M	€39.4 M	€40.6 M	€42.0 M	€43.5 M	€44.9 M	€46.4 M	€47.7 M
	Total	€112.3 M	€121.1 M	€133.5 M	€142.6 M	€149.5 M	€156.7 M	€161.7 M	€165.8 M
ADLIFE scenario	Primary care	€8.1 M	€9.2 M	€9.9 M	€10.3 M	€10.7 M	€11.0 M	€11.3 M	€11.4 M
	PC nurse	€4.4 M	€4.6 M	€4.9 M	€5.1 M	€5.3 M	€5.4 M	€5.6 M	€5.7 M
	PC doctor	€3.7 M	€4.6 M	€5.0 M	€5.2 M	€5.4 M	€5.5 M	€5.7 M	€5.7 M
	Hospital care	€61.1 M	€68.2 M	€78.7 M	€86.0 M	€90.9 M	€96.5 M	€100.1 M	€102.8 M
	Outpatient services	€9.4 M	€11.8 M	€13.5 M	€14.5 M	€15.5 M	€16.8 M	€17.8 M	€18.3 M
	Emergency room	€6.0 M	€7.5 M	€8.3 M	€9.2 M	€9.6 M	€10.0 M	€10.4 M	€10.8 M
	Hospitalisation	€45.7 M	€48.9 M	€56.9 M	€62.3 M	€65.8 M	€69.7 M	€71.9 M	€73.7 M
	Drug prescription	€38.4 M	€39.4 M	€40.6 M	€42.0 M	€43.5 M	€44.9 M	€46.4 M	€47.7 M
	Total	€107.5 M	€116.8 M	€129.2 M	€138.4 M	€145.1 M	€152.3 M	€157.8 M	€161.9 M
Difference		-€4.8 M	-€4.3 M	-€4.3 M	-€4.3 M	-€4.4 M	-€4.3 M	-€3.8 M	-€3.9 M

Table SM 37 - Budget impact analysis from 2023 to 2030 at the Germany pilot site.

		2023	2024	2025	2026	2027	2028	2029	2030
Conventional scenario	Primary care	€3.2 M	€3.4 M	€3.6 M	€3.7 M	€3.8 M	€3.9 M	€4.0 M	€4.0 M
	PC nurse	€1.5 M	€1.6 M	€1.7 M	€1.7 M	€1.8 M	€1.9 M	€1.9 M	€2.0 M
	PC doctor	€1.7 M	€1.8 M	€1.9 M	€2.0 M	€2.0 M	€2.1 M	€2.1 M	€2.1 M
	Hospital care	€7.1 M	€7.8 M	€8.7 M	€10.3 M	€10.4 M	€11.6 M	€11.8 M	€12.6 M
	Outpatient services	€0.4 M	€0.5 M	€0.6 M	€0.6 M	€0.6 M	€0.7 M	€0.7 M	€0.8 M
	Emergency room	€0.3 M	€0.4 M	€0.4 M	€0.4 M	€0.5 M	€0.5 M	€0.5 M	€0.5 M
	Hospitalisation	€6.4 M	€7.0 M	€7.7 M	€9.3 M	€9.3 M	€10.4 M	€10.6 M	€11.4 M
	Drug prescription	€3.8 M	€3.8 M	€4.0 M	€4.1 M	€4.2 M	€4.3 M	€4.5 M	€4.6 M
	Total	€14.1 M	€15.1 M	€16.3 M	€18.1 M	€18.4 M	€19.9 M	€20.3 M	€21.3 M
ADLIFE scenario	Primary care	€2.6 M	€3.0 M	€3.2 M	€3.3 M	€3.4 M	€3.5 M	€3.6 M	€3.6 M
	PC nurse	€1.4 M	€1.5 M	€1.6 M	€1.6 M	€1.7 M	€1.7 M	€1.8 M	€1.8 M
	PC doctor	€1.2 M	€1.5 M	€1.6 M	€1.7 M	€1.8 M	€1.8 M	€1.9 M	€1.8 M
	Hospital care	€7.0 M	€7.7 M	€8.6 M	€10.2 M	€10.3 M	€11.5 M	€11.8 M	€12.6 M
	Outpatient services	€0.4 M	€0.5 M	€0.6 M	€0.6 M	€0.7 M	€0.8 M	€0.8 M	€0.8 M
	Emergency room	€0.2 M	€0.2 M	€0.3 M	€0.3 M	€0.3 M	€0.3 M	€0.3 M	€0.4 M
	Hospitalisation	€6.4 M	€7.0 M	€7.7 M	€9.3 M	€9.3 M	€10.4 M	€10.6 M	€11.4 M
	Drug prescription	€3.8 M	€3.8 M	€4.0 M	€4.1 M	€4.2 M	€4.3 M	€4.5 M	€4.6 M
	Total	€13.4 M	€14.5 M	€15.8 M	€17.7 M	€17.9 M	€19.4 M	€19.9 M	€20.9 M
Difference		-€0.6 M	-€0.6 M	-€0.5 M	-€0.5 M	-€0.5 M	-€0.5 M	-€0.4 M	-€0.5 M

Table SM 38 - Budget impact analysis from 2023 to 2030 at the Scotland pilot site.

		2023	2024	2025	2026	2027	2028	2029	2030
Conventional scenario	Primary care	€8.9 M	€9.6 M	€10.0 M	€10.4 M	€10.7 M	€11.0 M	€11.2 M	€11.5 M
	PC nurse	€3.6 M	€3.9 M	€4.0 M	€4.2 M	€4.4 M	€4.6 M	€4.7 M	€4.9 M
	PC doctor	€5.2 M	€5.7 M	€5.9 M	€6.2 M	€6.3 M	€6.4 M	€6.5 M	€6.6 M
	Hospital care	€27.9 M	€30.8 M	€35.8 M	€39.5 M	€42.1 M	€43.5 M	€45.7 M	€48.0 M
	Outpatient services	€2.0 M	€2.5 M	€2.9 M	€3.2 M	€3.4 M	€3.7 M	€4.1 M	€4.2 M
	Emergency room	€2.6 M	€3.0 M	€3.3 M	€3.5 M	€3.7 M	€3.9 M	€4.0 M	€4.1 M
	Hospitalisation	€23.2 M	€25.3 M	€29.6 M	€32.8 M	€35.0 M	€35.9 M	€37.6 M	€39.7 M
	Drug prescription	€17.9 M	€18.4 M	€19.1 M	€19.8 M	€20.5 M	€21.3 M	€22.0 M	€22.9 M
	Total	€54.6 M	€58.8 M	€64.8 M	€69.6 M	€73.3 M	€75.8 M	€78.9 M	€82.4 M
ADLIFE scenario	Primary care	€7.3 M	€8.4 M	€9.0 M	€9.4 M	€9.8 M	€10.1 M	€10.4 M	€10.6 M
	PC nurse	€3.5 M	€3.8 M	€4.0 M	€4.1 M	€4.3 M	€4.5 M	€4.6 M	€4.7 M
	PC doctor	€3.7 M	€4.6 M	€5.0 M	€5.3 M	€5.5 M	€5.7 M	€5.8 M	€5.9 M
	Hospital care	€27.1 M	€30.1 M	€35.0 M	€38.7 M	€41.4 M	€42.8 M	€45.0 M	€47.4 M
	Outpatient services	€2.3 M	€2.9 M	€3.3 M	€3.5 M	€3.8 M	€4.1 M	€4.5 M	€4.7 M
	Emergency room	€1.6 M	€2.0 M	€2.1 M	€2.4 M	€2.5 M	€2.7 M	€2.8 M	€2.9 M
	Hospitalisation	€23.2 M	€25.3 M	€29.6 M	€32.8 M	€35.0 M	€35.9 M	€37.6 M	€39.7 M
	Drug prescription	€17.9 M	€18.4 M	€19.1 M	€19.8 M	€20.5 M	€21.3 M	€22.0 M	€22.9 M
	Total	€52.3 M	€56.9 M	€63.1 M	€67.9 M	€71.7 M	€74.2 M	€77.4 M	€80.9 M
Difference		-€2.3 M	-€1.9 M	-€1.8 M	-€1.7 M	-€1.6 M	-€1.6 M	-€1.6 M	-€1.5 M

Table SM 39 - C3-CLOUD intervention effect presented as odd ratio and significance.

Resource	C3-CLOUD intervention effect
PC nurse at centre[a]	0,74 (0,57-0,96)*
PC nurse by telephone[a]	1,60 (1,15-2,23)**
PC nurse at home[a]	0,45 (0,23-0,86)*
PC doctor at centre[a]	0,63 (0,44-0,89)**
Emergency room[b]	0,57 (0,35-0,93)*

[a] Calculated using generalized linear model (family:negative binomial, link:log).

[b] Calculated using logistic regression.

* p-value ≤ 0.05, ** p-value ≤ 0.01.

Table SM 40 - Budget impact analysis from 2023 to 2030 for the Scenario 1.

		2023	2024	2025	2026	2027	2028	2029	2030
Conventional scenario	Primary care	€22.2 M	€23.9 M	€25.0 M	€25.9 M	€26.5 M	€27.0 M	€27.4 M	€27.9 M
	PC nurse	€7.7 M	€7.9 M	€8.2 M	€8.6 M	€8.9 M	€9.1 M	€9.3 M	€9.6 M
	PC doctor	€14.5 M	€16.0 M	€16.7 M	€17.3 M	€17.6 M	€17.9 M	€18.1 M	€18.3 M
	Hospital care	€116.3 M	€129.6 M	€144.5 M	€156.6 M	€169.2 M	€174.5 M	€181.6 M	€186.5 M
	Outpatient services	€6.6 M	€8.3 M	€9.5 M	€10.3 M	€11.0 M	€12.1 M	€12.9 M	€13.6 M
	Emergency room	€13.8 M	€15.9 M	€17.3 M	€18.3 M	€19.2 M	€19.6 M	€20.1 M	€20.7 M
	Hospitalisation	€95.9 M	€105.4 M	€117.7 M	€128.0 M	€138.9 M	€142.8 M	€148.6 M	€152.3 M
	Drug prescription	€75.6 M	€78.0 M	€80.6 M	€83.7 M	€87.0 M	€90.1 M	€93.3 M	€96.6 M
	Total	€214.1 M	€231.5 M	€250.1 M	€266.2 M	€282.7 M	€291.6 M	€302.3 M	€311.1 M
ADLIFE scenario	Primary care	€20.5 M	€22.8 M	€24.0 M	€25.0 M	€25.7 M	€26.2 M	€26.6 M	€27.1 M
	PC nurse	€7.6 M	€7.8 M	€8.1 M	€8.5 M	€8.8 M	€9.0 M	€9.2 M	€9.4 M
	PC doctor	€12.9 M	€15.0 M	€15.9 M	€16.5 M	€16.9 M	€17.2 M	€17.4 M	€17.7 M
	Hospital care	€114.6 M	€128.0 M	€143.1 M	€155.3 M	€167.9 M	€173.3 M	€180.6 M	€185.4 M
	Outpatient services	€7.6 M	€9.3 M	€10.6 M	€11.5 M	€12.4 M	€13.5 M	€14.5 M	€15.2 M
	Emergency room	€11.2 M	€13.3 M	€14.7 M	€15.8 M	€16.6 M	€17.0 M	€17.5 M	€17.9 M
	Hospitalisation	€95.9 M	€105.4 M	€117.7 M	€128.0 M	€138.9 M	€142.8 M	€148.6 M	€152.3 M
	Drug prescription	€75.6 M	€78.0 M	€80.6 M	€83.7 M	€87.0 M	€90.1 M	€93.3 M	€96.6 M
	Total	€210.8 M	€228.8 M	€247.7 M	€263.9 M	€280.5 M	€289.6 M	€300.5 M	€309.1 M
Difference		-€3.4 M	-€2.7 M	-€2.4 M	-€2.2 M	-€2.2 M	-€2.0 M	-€1.8 M	-€1.9 M

Table SM 41 - Budget impact analysis from 2023 to 2030 for the Scenario 2.

		2023	2024	2025	2026	2027	2028	2029	2030
Conventional scenario	Primary care	€22.2 M	€23.9 M	€25.0 M	€25.9 M	€26.5 M	€27.1 M	€27.4 M	€27.8 M
	PC nurse	€7.7 M	€7.9 M	€8.2 M	€8.6 M	€8.9 M	€9.1 M	€9.3 M	€9.6 M
	PC doctor	€14.5 M	€16.0 M	€16.7 M	€17.2 M	€17.7 M	€17.9 M	€18.0 M	€18.3 M
	Hospital care	€116.3 M	€129.7 M	€144.0 M	€156.3 M	€168.3 M	€175.9 M	€181.9 M	€187.2 M
	Outpatient services	€6.6 M	€8.3 M	€9.5 M	€10.4 M	€11.1 M	€12.1 M	€12.9 M	€13.4 M
	Emergency room	€13.8 M	€15.9 M	€17.3 M	€18.3 M	€19.2 M	€19.6 M	€20.2 M	€20.7 M
	Hospitalisation	€95.9 M	€105.5 M	€117.3 M	€127.6 M	€138.0 M	€144.2 M	€148.7 M	€153.1 M
	Drug prescription	€75.6 M	€78.0 M	€80.6 M	€83.6 M	€86.9 M	€90.1 M	€93.2 M	€96.3 M
	Total	€214.1 M	€231.6 M	€249.6 M	€265.8 M	€281.6 M	€293.1 M	€302.4 M	€311.4 M
ADLIFE scenario	Primary care	€18.1 M	€20.8 M	€22.3 M	€23.3 M	€24.1 M	€24.7 M	€25.1 M	€25.5 M
	PC nurse	€7.5 M	€7.7 M	€8.0 M	€8.4 M	€8.6 M	€8.8 M	€9.0 M	€9.3 M
	PC doctor	€10.6 M	€13.1 M	€14.3 M	€15.0 M	€15.5 M	€15.8 M	€16.1 M	€16.2 M
	Hospital care	€112.1 M	€125.7 M	€139.9 M	€152.2 M	€164.2 M	€172.0 M	€178.0 M	€183.3 M
	Outpatient services	€8.0 M	€9.8 M	€11.1 M	€12.1 M	€13.0 M	€14.1 M	€15.1 M	€15.8 M
	Emergency room	€8.3 M	€10.4 M	€11.5 M	€12.5 M	€13.2 M	€13.7 M	€14.1 M	€14.4 M
	Hospitalisation	€95.9 M	€105.5 M	€117.3 M	€127.6 M	€138.0 M	€144.2 M	€148.7 M	€153.1 M
	Drug prescription	€75.6 M	€78.0 M	€80.6 M	€83.6 M	€86.9 M	€90.1 M	€93.2 M	€96.3 M
	Total	€205.9 M	€224.5 M	€242.8 M	€259.2 M	€275.2 M	€286.9 M	€296.3 M	€305.2 M
Difference		-€8.3 M	-€7.2 M	-€6.8 M	-€6.6 M	-€6.5 M	-€6.2 M	-€6.2 M	-€6.3 M

Table SM 42 - Budget impact analysis from 2023 to 2030 for the Scenario 3.

		2023	2024	2025	2026	2027	2028	2029	2030
Conventional scenario	Primary care	€22.2 M	€23.9 M	€25.0 M	€25.9 M	€26.5 M	€27.0 M	€27.3 M	€27.8 M
	PC nurse	€7.7 M	€7.9 M	€8.2 M	€8.6 M	€8.9 M	€9.1 M	€9.3 M	€9.6 M
	PC doctor	€14.5 M	€16.0 M	€16.7 M	€17.2 M	€17.6 M	€17.9 M	€18.1 M	€18.3 M
	Hospital care	€116.3 M	€129.6 M	€144.3 M	€156.2 M	€169.3 M	€175.2 M	€181.2 M	€186.3 M
	Outpatient services	€6.6 M	€8.3 M	€9.5 M	€10.3 M	€11.0 M	€12.1 M	€12.9 M	€13.5 M
	Emergency room	€13.8 M	€15.9 M	€17.3 M	€18.3 M	€19.1 M	€19.6 M	€20.1 M	€20.6 M
	Hospitalisation	€95.9 M	€105.3 M	€117.5 M	€127.6 M	€139.2 M	€143.5 M	€148.3 M	€152.2 M
	Drug prescription	€75.6 M	€78.0 M	€80.6 M	€83.5 M	€86.8 M	€90.0 M	€93.0 M	€96.4 M
	Total	€214.1 M	€231.5 M	€249.9 M	€265.6 M	€282.6 M	€292.3 M	€301.5 M	€310.5 M
ADLIFE scenario	Primary care	€20.5 M	€22.8 M	€24.0 M	€25.0 M	€25.7 M	€26.2 M	€26.5 M	€27.1 M
	PC nurse	€7.6 M	€7.8 M	€8.1 M	€8.5 M	€8.8 M	€9.0 M	€9.1 M	€9.4 M
	PC doctor	€12.9 M	€15.0 M	€15.9 M	€16.4 M	€16.9 M	€17.2 M	€17.4 M	€17.6 M
	Hospital care	€115.0 M	€128.5 M	€143.4 M	€155.4 M	€168.6 M	€174.6 M	€180.9 M	€185.8 M
	Outpatient services	€8.0 M	€9.8 M	€11.1 M	€12.1 M	€12.9 M	€14.1 M	€15.1 M	€15.8 M
	Emergency room	€11.2 M	€13.3 M	€14.8 M	€15.8 M	€16.5 M	€17.0 M	€17.5 M	€17.8 M
	Hospitalisation	€95.9 M	€105.3 M	€117.5 M	€127.6 M	€139.2 M	€143.5 M	€148.3 M	€152.2 M
	Drug prescription	€75.6 M	€78.0 M	€80.6 M	€83.5 M	€86.8 M	€90.0 M	€93.0 M	€96.4 M
	Total	€211.2 M	€229.3 M	€248.0 M	€263.9 M	€281.1 M	€290.9 M	€300.4 M	€309.2 M
Difference		-€2.9 M	-€2.2 M	-€1.8 M	-€1.7 M	-€1.5 M	-€1.4 M	-€1.1 M	-€1.3 M

Table SM 43 - Budget impact analysis from 2023 to 2030 for the Scenario 4.

		2023	2024	2025	2026	2027	2028	2029	2030
Conventional scenario	Primary care	€28.9 M	€31.2 M	€32.6 M	€33.6 M	€34.4 M	€35.0 M	€35.5 M	€36.0 M
	PC nurse	€10.1 M	€10.3 M	€10.8 M	€11.2 M	€11.5 M	€11.8 M	€12.1 M	€12.3 M
	PC doctor	€18.8 M	€20.9 M	€21.8 M	€22.4 M	€22.8 M	€23.2 M	€23.4 M	€23.7 M
	Hospital care	€150.3 M	€167.2 M	€187.8 M	€204.4 M	€217.9 M	€231.0 M	€237.5 M	€244.8 M
	Outpatient services	€8.6 M	€10.8 M	€12.3 M	€13.3 M	€14.3 M	€15.7 M	€16.8 M	€17.4 M
	Emergency room	€17.9 M	€20.6 M	€22.5 M	€24.0 M	€25.1 M	€25.6 M	€26.4 M	€26.9 M
	Hospitalisation	€123.8 M	€135.9 M	€153.1 M	€167.0 M	€178.5 M	€189.7 M	€194.3 M	€200.5 M
	Drug prescription	€98.2 M	€101.4 M	€105.1 M	€108.9 M	€112.9 M	€117.0 M	€120.8 M	€124.9 M
	Total	€277.4 M	€299.8 M	€325.5 M	€346.9 M	€365.2 M	€383.0 M	€393.9 M	€405.7 M
ADLIFE scenario	Primary care	€26.7 M	€29.7 M	€31.3 M	€32.5 M	€33.3 M	€33.9 M	€34.5 M	€34.9 M
	PC nurse	€9.9 M	€10.2 M	€10.6 M	€11.0 M	€11.4 M	€11.6 M	€11.9 M	€12.1 M
	PC doctor	€16.8 M	€19.5 M	€20.7 M	€21.4 M	€21.9 M	€22.3 M	€22.6 M	€22.8 M
	Hospital care	€148.7 M	€165.8 M	€186.8 M	€203.1 M	€217.0 M	€230.2 M	€237.0 M	€244.4 M
	Outpatient services	€10.4 M	€12.7 M	€14.4 M	€15.6 M	€16.8 M	€18.3 M	€19.7 M	€20.5 M
	Emergency room	€14.5 M	€17.2 M	€19.2 M	€20.4 M	€21.8 M	€22.2 M	€22.9 M	€23.4 M
	Hospitalisation	€123.8 M	€135.9 M	€153.1 M	€167.0 M	€178.5 M	€189.7 M	€194.3 M	€200.5 M
	Drug prescription	€98.2 M	€101.4 M	€105.1 M	€108.9 M	€112.9 M	€117.0 M	€120.8 M	€124.9 M
	Total	€273.6 M	€296.9 M	€323.1 M	€344.5 M	€363.2 M	€381.1 M	€392.3 M	€404.2 M
Difference		-€3.8 M	-€2.9 M	-€2.3 M	-€2.4 M	-€2.0 M	-€1.8 M	-€1.6 M	-€1.4 M

Table SM 44 - Budget impact analysis from 2023 to 2030 for the Scenario 5.

		2023	2024	2025	2026	2027	2028	2029	2030
Conventional scenario	Primary care	€15.6 M	€16.8 M	€17.5 M	€18.1 M	€18.5 M	€18.9 M	€19.3 M	€19.6 M
	PC nurse	€5.4 M	€5.6 M	€5.8 M	€6.0 M	€6.2 M	€6.4 M	€6.6 M	€6.7 M
	PC doctor	€10.2 M	€11.2 M	€11.7 M	€12.1 M	€12.3 M	€12.5 M	€12.7 M	€12.9 M
	Hospital care	€81.2 M	€91.0 M	€101.7 M	€110.4 M	€118.2 M	€121.6 M	€128.0 M	€132.1 M
	Outpatient services	€4.7 M	€5.8 M	€6.7 M	€7.2 M	€7.7 M	€8.4 M	€9.1 M	€9.5 M
	Emergency room	€9.7 M	€11.1 M	€12.2 M	€13.0 M	€13.4 M	€13.8 M	€14.2 M	€14.5 M
	Hospitalisation	€66.9 M	€74.1 M	€82.9 M	€90.2 M	€97.0 M	€99.4 M	€104.7 M	€108.2 M
	Drug prescription	€52.9 M	€54.6 M	€56.6 M	€58.8 M	€61.0 M	€62.9 M	€65.3 M	€67.6 M
	Total	€149.8 M	€162.4 M	€175.7 M	€187.4 M	€197.7 M	€203.4 M	€212.5 M	€219.2 M
ADLIFE scenario	Primary care	€14.4 M	€16.0 M	€16.8 M	€17.5 M	€17.9 M	€18.3 M	€18.7 M	€19.0 M
	PC nurse	€5.4 M	€5.5 M	€5.7 M	€5.9 M	€6.1 M	€6.3 M	€6.5 M	€6.6 M
	PC doctor	€9.1 M	€10.5 M	€11.2 M	€11.6 M	€11.8 M	€12.0 M	€12.2 M	€12.4 M
	Hospital care	€80.4 M	€90.3 M	€101.1 M	€109.8 M	€117.8 M	€121.2 M	€127.6 M	€131.8 M
	Outpatient services	€5.6 M	€6.9 M	€7.8 M	€8.5 M	€9.1 M	€9.9 M	€10.6 M	€11.1 M
	Emergency room	€7.9 M	€9.3 M	€10.3 M	€11.2 M	€11.6 M	€12.0 M	€12.4 M	€12.6 M
	Hospitalisation	€66.9 M	€74.1 M	€82.9 M	€90.2 M	€97.0 M	€99.4 M	€104.7 M	€108.2 M
	Drug prescription	€52.9 M	€54.6 M	€56.6 M	€58.8 M	€61.0 M	€62.9 M	€65.3 M	€67.6 M
	Total	€147.7 M	€160.9 M	€174.5 M	€186.1 M	€196.7 M	€202.5 M	€211.7 M	€218.4 M
Difference		-€2.1 M	-€1.5 M	-€1.3 M	-€1.3 M	-€1.0 M	-€0.9 M	-€0.9 M	-€0.9 M

Table SM 45 - Budget impact analysis from 2023 to 2030 for the Scenario 6.

		2023	2024	2025	2026	2027	2028	2029	2030
Conventional scenario	Primary care	€34.5 M	€37.2 M	€38.7 M	€40.1 M	€41.2 M	€41.9 M	€42.6 M	€43.6 M
	PC nurse	€12.9 M	€13.1 M	€13.8 M	€14.4 M	€14.9 M	€15.4 M	€15.8 M	€16.3 M
	PC doctor	€21.7 M	€24.1 M	€25.0 M	€25.7 M	€26.3 M	€26.5 M	€26.8 M	€27.3 M
	Hospital care	€172.1 M	€205.4 M	€232.1 M	€251.4 M	€265.8 M	€278.2 M	€291.6 M	€301.6 M
	Outpatient services	€10.2 M	€13.2 M	€15.1 M	€16.4 M	€17.5 M	€19.3 M	€20.6 M	€21.7 M
	Emergency room	€20.9 M	€24.9 M	€27.4 M	€28.7 M	€30.1 M	€30.9 M	€31.5 M	€31.9 M
	Hospitalisation	€141.0 M	€167.3 M	€189.6 M	€206.4 M	€218.2 M	€228.0 M	€239.5 M	€248.1 M
	Drug prescription	€75.6 M	€78.0 M	€80.6 M	€83.7 M	€86.9 M	€90.2 M	€93.3 M	€96.5 M
	Total	€282.3 M	€320.6 M	€351.5 M	€375.2 M	€393.9 M	€410.3 M	€427.5 M	€441.7 M
ADLIFE scenario	Primary care	€32.4 M	€35.9 M	€37.7 M	€39.1 M	€40.2 M	€41.0 M	€41.7 M	€42.6 M
	PC nurse	€12.7 M	€12.9 M	€13.6 M	€14.2 M	€14.7 M	€15.2 M	€15.6 M	€16.1 M
	PC doctor	€19.7 M	€23.0 M	€24.1 M	€24.9 M	€25.5 M	€25.8 M	€26.1 M	€26.6 M
	Hospital care	€170.3 M	€203.9 M	€230.7 M	€250.3 M	€264.8 M	€277.3 M	€291.3 M	€301.3 M
	Outpatient services	€12.2 M	€15.3 M	€17.5 M	€18.9 M	€20.2 M	€22.2 M	€23.7 M	€24.9 M
	Emergency room	€17.1 M	€21.2 M	€23.5 M	€25.0 M	€26.4 M	€27.1 M	€28.1 M	€28.3 M
	Hospitalisation	€141.0 M	€167.3 M	€189.6 M	€206.4 M	€218.2 M	€228.0 M	€239.5 M	€248.1 M
	Drug prescription	€75.6 M	€78.0 M	€80.6 M	€83.7 M	€86.9 M	€90.2 M	€93.3 M	€96.5 M
	Total	€278.4 M	€317.8 M	€349.0 M	€373.1 M	€391.9 M	€408.6 M	€426.2 M	€440.5 M
Difference		-€3.9 M	-€2.8 M	-€2.5 M	-€2.1 M	-€2.0 M	-€1.7 M	-€1.3 M	-€1.2 M

Table SM 46 - Budget impact analysis from 2023 to 2030 for the Scenario 7.

		2023	2024	2025	2026	2027	2028	2029	2030
Conventional scenario	Primary care	€12.4 M	€13.2 M	€13.8 M	€14.3 M	€14.7 M	€15.0 M	€15.2 M	€15.4 M
	PC nurse	€4.0 M	€4.0 M	€4.1 M	€4.3 M	€4.4 M	€4.5 M	€4.6 M	€4.7 M
	PC doctor	€8.4 M	€9.2 M	€9.7 M	€10.0 M	€10.3 M	€10.5 M	€10.6 M	€10.8 M
	Hospital care	€69.8 M	€72.2 M	€77.4 M	€83.5 M	€89.5 M	€92.8 M	€97.4 M	€101.6 M
	Outpatient services	€3.8 M	€4.4 M	€5.0 M	€5.5 M	€5.9 M	€6.4 M	€6.8 M	€7.2 M
	Emergency room	€8.1 M	€8.8 M	€9.5 M	€9.9 M	€10.6 M	€10.8 M	€11.0 M	€11.3 M
	Hospitalisation	€57.9 M	€59.0 M	€62.8 M	€68.1 M	€73.1 M	€75.7 M	€79.6 M	€83.0 M
	Drug prescription	€75.6 M	€78.0 M	€80.6 M	€83.5 M	€86.9 M	€90.1 M	€93.1 M	€96.3 M
	Total	€157.9 M	€163.3 M	€171.8 M	€181.3 M	€191.1 M	€198.0 M	€205.8 M	€213.3 M
ADLIFE scenario	Primary care	€11.2 M	€12.2 M	€13.0 M	€13.6 M	€14.0 M	€14.3 M	€14.6 M	€14.7 M
	PC nurse	€3.9 M	€3.9 M	€4.1 M	€4.2 M	€4.3 M	€4.4 M	€4.5 M	€4.6 M
	PC doctor	€7.3 M	€8.3 M	€9.0 M	€9.4 M	€9.7 M	€9.9 M	€10.0 M	€10.1 M
	Hospital care	€69.1 M	€71.5 M	€76.8 M	€83.1 M	€88.9 M	€92.4 M	€97.1 M	€101.2 M
	Outpatient services	€4.6 M	€5.3 M	€6.1 M	€6.6 M	€7.0 M	€7.6 M	€8.2 M	€8.6 M
	Emergency room	€6.5 M	€7.3 M	€7.9 M	€8.4 M	€8.9 M	€9.2 M	€9.3 M	€9.5 M
	Hospitalisation	€57.9 M	€59.0 M	€62.8 M	€68.1 M	€73.1 M	€75.7 M	€79.6 M	€83.0 M
	Drug prescription	€75.6 M	€78.0 M	€80.6 M	€83.5 M	€86.9 M	€90.1 M	€93.1 M	€96.3 M
	Total	€155.9 M	€161.8 M	€170.4 M	€180.2 M	€189.8 M	€196.9 M	€204.8 M	€212.2 M
Difference		-€1.9 M	-€1.6 M	-€1.4 M	-€1.1 M	-€1.3 M	-€1.1 M	-€1.0 M	-€1.1 M

Table SM 47 - Budget impact analysis from 2023 to 2030 for the Scenario 8.

		2023	2024	2025	2026	2027	2028	2029	2030
Conventional scenario	Primary care	€28.9 M	€31.1 M	€32.5 M	€33.6 M	€34.5 M	€35.1 M	€35.5 M	€36.2 M
	PC nurse	€10.1 M	€10.3 M	€10.7 M	€11.2 M	€11.5 M	€11.8 M	€12.1 M	€12.5 M
	PC doctor	€18.8 M	€20.8 M	€21.8 M	€22.4 M	€22.9 M	€23.3 M	€23.5 M	€23.7 M
	Hospital care	€151.2 M	€168.4 M	€187.6 M	€203.1 M	€220.1 M	€227.8 M	€235.6 M	€242.1 M
	Outpatient services	€8.6 M	€10.8 M	€12.3 M	€13.4 M	€14.3 M	€15.7 M	€16.7 M	€17.5 M
	Emergency room	€17.9 M	€20.7 M	€22.4 M	€23.8 M	€24.8 M	€25.5 M	€26.1 M	€26.8 M
	Hospitalisation	€124.6 M	€137.0 M	€152.8 M	€165.8 M	€181.0 M	€186.6 M	€192.8 M	€197.8 M
	Drug prescription	€75.6 M	€78.0 M	€80.6 M	€83.5 M	€86.8 M	€90.0 M	€93.0 M	€96.4 M
	Total	€255.7 M	€277.5 M	€300.6 M	€320.3 M	€341.4 M	€353.0 M	€364.1 M	€374.7 M
ADLIFE scenario	Primary care	€26.7 M	€29.6 M	€31.2 M	€32.5 M	€33.4 M	€34.1 M	€34.5 M	€35.2 M
	PC nurse	€9.9 M	€10.1 M	€10.6 M	€11.1 M	€11.4 M	€11.7 M	€11.9 M	€12.3 M
	PC doctor	€16.8 M	€19.5 M	€20.6 M	€21.4 M	€22.0 M	€22.4 M	€22.6 M	€22.9 M
	Hospital care	€149.5 M	€167.0 M	€186.5 M	€202.1 M	€219.2 M	€227.0 M	€235.2 M	€241.5 M
	Outpatient services	€10.4 M	€12.7 M	€14.5 M	€15.7 M	€16.8 M	€18.3 M	€19.7 M	€20.5 M
	Emergency room	€14.5 M	€17.3 M	€19.2 M	€20.5 M	€21.4 M	€22.1 M	€22.7 M	€23.2 M
	Hospitalisation	€124.6 M	€137.0 M	€152.8 M	€165.8 M	€181.0 M	€186.6 M	€192.8 M	€197.8 M
	Drug prescription	€75.6 M	€78.0 M	€80.6 M	€83.5 M	€86.8 M	€90.0 M	€93.0 M	€96.4 M
	Total	€251.9 M	€274.7 M	€298.3 M	€318.0 M	€339.4 M	€351.2 M	€362.6 M	€373.1 M
Difference		-€3.8 M	-€2.9 M	-€2.4 M	-€2.2 M	-€2.0 M	-€1.8 M	-€1.5 M	-€1.6 M

Table SM 48 - Budget impact analysis from 2023 to 2030 for the Scenario 9.

		2023	2024	2025	2026	2027	2028	2029	2030
Conventional scenario	Primary care	€15.6 M	€16.7 M	€17.5 M	€18.1 M	€18.6 M	€18.9 M	€19.1 M	€19.5 M
	PC nurse	€5.4 M	€5.5 M	€5.8 M	€6.0 M	€6.2 M	€6.4 M	€6.5 M	€6.7 M
	PC doctor	€10.1 M	€11.2 M	€11.7 M	€12.1 M	€12.3 M	€12.5 M	€12.6 M	€12.8 M
	Hospital care	€81.4 M	€90.7 M	€101.0 M	€109.4 M	€118.5 M	€122.7 M	€126.9 M	€130.4 M
	Outpatient services	€4.6 M	€5.8 M	€6.6 M	€7.2 M	€7.7 M	€8.4 M	€9.0 M	€9.4 M
	Emergency room	€9.6 M	€11.1 M	€12.1 M	€12.8 M	€13.3 M	€13.7 M	€14.1 M	€14.4 M
	Hospitalisation	€67.1 M	€73.7 M	€82.3 M	€89.3 M	€97.5 M	€100.5 M	€103.8 M	€106.5 M
	Drug prescription	€75.6 M	€78.0 M	€80.6 M	€83.5 M	€86.8 M	€90.0 M	€93.0 M	€96.4 M
	Total	€172.6 M	€185.4 M	€199.1 M	€211.0 M	€223.9 M	€231.6 M	€238.9 M	€246.2 M
ADLIFE scenario	Primary care	€14.4 M	€16.0 M	€16.8 M	€17.5 M	€18.0 M	€18.3 M	€18.6 M	€18.9 M
	PC nurse	€5.4 M	€5.4 M	€5.7 M	€6.0 M	€6.1 M	€6.3 M	€6.4 M	€6.6 M
	PC doctor	€9.0 M	€10.5 M	€11.1 M	€11.5 M	€11.9 M	€12.1 M	€12.2 M	€12.3 M
	Hospital care	€80.5 M	€89.9 M	€100.4 M	€108.8 M	€118.0 M	€122.2 M	€126.6 M	€130.1 M
	Outpatient services	€5.6 M	€6.9 M	€7.8 M	€8.5 M	€9.0 M	€9.9 M	€10.6 M	€11.0 M
	Emergency room	€7.8 M	€9.3 M	€10.3 M	€11.0 M	€11.5 M	€11.9 M	€12.2 M	€12.5 M
	Hospitalisation	€67.1 M	€73.7 M	€82.3 M	€89.3 M	€97.5 M	€100.5 M	€103.8 M	€106.5 M
	Drug prescription	€75.6 M	€78.0 M	€80.6 M	€83.5 M	€86.8 M	€90.0 M	€93.0 M	€96.4 M
	Total	€170.5 M	€183.9 M	€197.8 M	€209.8 M	€222.8 M	€230.6 M	€238.1 M	€245.4 M
Difference		-€2.1 M	-€1.5 M	-€1.3 M	-€1.2 M	-€1.1 M	-€1.0 M	-€0.8 M	-€0.9 M

Table SM 49 - Budget impact analysis from 2023 to 2030 for the Scenario 10.

		2023	2024	2025	2026	2027	2028	2029	2030
Conventional scenario	Primary care	€18.3 M	€19.6 M	€20.5 M	€21.3 M	€21.8 M	€22.2 M	€22.5 M	€22.9 M
	PC nurse	€7.6 M	€7.8 M	€8.1 M	€8.5 M	€8.7 M	€8.9 M	€9.1 M	€9.4 M
	PC doctor	€10.7 M	€11.9 M	€12.4 M	€12.8 M	€13.1 M	€13.3 M	€13.4 M	€13.5 M
	Hospital care	€113.2 M	€126.1 M	€140.6 M	€152.4 M	€165.3 M	€171.3 M	€177.3 M	€182.3 M
	Outpatient services	€7.7 M	€9.6 M	€11.0 M	€12.0 M	€12.8 M	€14.0 M	€15.0 M	€15.7 M
	Emergency room	€9.6 M	€11.1 M	€12.1 M	€12.8 M	€13.3 M	€13.7 M	€14.1 M	€14.4 M
	Hospitalisation	€95.9 M	€105.3 M	€117.5 M	€127.6 M	€139.2 M	€143.5 M	€148.3 M	€152.2 M
	Drug prescription	€75.6 M	€78.0 M	€80.6 M	€83.5 M	€86.8 M	€90.0 M	€93.0 M	€96.4 M
	Total	€207.1 M	€223.7 M	€241.7 M	€257.2 M	€274.0 M	€283.6 M	€292.8 M	€301.6 M
ADLIFE scenario	Primary care	€17.1 M	€18.9 M	€19.9 M	€20.7 M	€21.2 M	€21.7 M	€21.9 M	€22.3 M
	PC nurse	€7.6 M	€7.7 M	€8.0 M	€8.4 M	€8.6 M	€8.8 M	€9.0 M	€9.3 M
	PC doctor	€9.5 M	€11.2 M	€11.9 M	€12.3 M	€12.6 M	€12.8 M	€12.9 M	€13.1 M
	Hospital care	€113.1 M	€126.2 M	€141.0 M	€152.9 M	€166.0 M	€172.1 M	€178.4 M	€183.4 M
	Outpatient services	€9.4 M	€11.5 M	€13.1 M	€14.3 M	€15.2 M	€16.7 M	€17.9 M	€18.7 M
	Emergency room	€7.8 M	€9.3 M	€10.3 M	€11.0 M	€11.5 M	€11.9 M	€12.2 M	€12.5 M
	Hospitalisation	€95.9 M	€105.3 M	€117.5 M	€127.6 M	€139.2 M	€143.5 M	€148.3 M	€152.2 M
	Drug prescription	€75.6 M	€78.0 M	€80.6 M	€83.5 M	€86.8 M	€90.0 M	€93.0 M	€96.4 M
	Total	€205.8 M	€223.1 M	€241.4 M	€257.1 M	€274.0 M	€283.8 M	€293.3 M	€302.1 M
Difference		-€1.3 M	-€0.6 M	-€0.3 M	-€0.1 M	€0.0 M	€0.3 M	€0.6 M	€0.5 M

Table SM 50 - Budget impact analysis from 2023 to 2030 for the Scenario 11.

		2023	2024	2025	2026	2027	2028	2029	2030
Conventional scenario	Primary care	€22.2 M	€23.9 M	€25.0 M	€25.9 M	€26.5 M	€27.0 M	€27.4 M	€27.8 M
	PC nurse	€7.7 M	€7.9 M	€8.2 M	€8.6 M	€8.9 M	€9.1 M	€9.3 M	€9.5 M
	PC doctor	€14.5 M	€16.0 M	€16.7 M	€17.2 M	€17.6 M	€17.9 M	€18.0 M	€18.3 M
	Hospital care	€116.3 M	€129.8 M	€144.0 M	€156.2 M	€168.8 M	€175.0 M	€181.5 M	€187.5 M
	Outpatient services	€6.6 M	€8.3 M	€9.5 M	€10.3 M	€11.0 M	€12.1 M	€12.9 M	€13.5 M
	Emergency room	€13.8 M	€15.9 M	€17.3 M	€18.4 M	€19.2 M	€19.6 M	€20.1 M	€20.5 M
	Hospitalisation	€95.9 M	€105.6 M	€117.3 M	€127.6 M	€138.6 M	€143.4 M	€148.4 M	€153.5 M
	Drug prescription	€75.6 M	€78.0 M	€80.6 M	€83.5 M	€86.8 M	€90.0 M	€93.1 M	€96.4 M
	Total	€214.1 M	€231.7 M	€249.6 M	€265.6 M	€282.0 M	€292.0 M	€302.0 M	€311.7 M
ADLIFE scenario	Primary care	€17.7 M	€20.4 M	€21.8 M	€22.7 M	€23.3 M	€23.9 M	€24.2 M	€24.6 M
	PC nurse	€5.4 M	€5.8 M	€6.2 M	€6.5 M	€6.7 M	€6.9 M	€7.0 M	€7.2 M
	PC doctor	€12.3 M	€14.6 M	€15.6 M	€16.2 M	€16.6 M	€17.0 M	€17.2 M	€17.4 M
	Hospital care	€111.0 M	€124.6 M	€138.5 M	€150.7 M	€163.1 M	€169.5 M	€175.8 M	€181.7 M
	Outpatient services	€6.6 M	€8.3 M	€9.5 M	€10.3 M	€11.0 M	€12.1 M	€12.9 M	€13.5 M
	Emergency room	€8.5 M	€10.7 M	€11.8 M	€12.8 M	€13.5 M	€14.0 M	€14.4 M	€14.7 M
	Hospitalisation	€95.9 M	€105.6 M	€117.3 M	€127.6 M	€138.6 M	€143.4 M	€148.4 M	€153.5 M
	Drug prescription	€75.6 M	€78.0 M	€80.6 M	€83.5 M	€86.8 M	€90.0 M	€93.1 M	€96.4 M
	Total	€204.3 M	€223.0 M	€240.9 M	€256.9 M	€273.2 M	€283.3 M	€293.2 M	€302.6 M
Difference		-€9.8 M	-€8.7 M	-€8.7 M	-€8.7 M	-€8.8 M	-€8.6 M	-€8.8 M	-€9.1 M

Technical Note SM 1 - Mathematical expressions of the time-to-event functions based on the resulting distributions.

Gompertz and Weibull distributions were selected to model time-to-event functions and can be expressed with the formulas below[1].

$$Time\ to\ event\ (Gompertz) = \frac{1}{\beta} * ln\left(1 - \frac{\beta}{HR * \alpha} * ln(u)\right)$$

$$Time\ to\ event\ (Weibull) = \left(-\frac{ln(u)}{HR * \alpha}\right)^{\frac{1}{\beta}}$$

Where:

$$ln(\alpha) = x_0 + x_1 * sex + x_2 * age\ group + x_3 * hf + x_4 * copd + x_5 * Charlson\ group$$

The equations included a uniformly distributed random factor between 0 and 1 (u) and two parameters α and β that defined the characteristics of the distribution. The hazard ratio (HR) is used to incorporate intervention effect into the model.

[1] Román R, Comas M, Hoffmeister L, Castells X. Determining the lifetime density function using a continuous approach. J Epidemiol Community Health. 2007;61:923-5

Technical Note SM 2 - Method to approximate regression model outcome to hazard ratio.

Hazard ratios (HRs) were derived from the outcomes of the regression models for subsequent incorporation into the simulation model, allowing for a distinction between the ADLIFE scenario and the current scenario.

On the one hand, when using logistic regression and the modelled event is rare, the HR can be approximated by the resulting odd ratio (OR). However, when the studied event is not rare, these measures diverge. In such cases, established formulas can be used to approximate the difference between an OR and an HR. A commonly used correction formula is as follows[2]:

$$HR = \frac{OR}{1 - P_0 + (P_0 * \ OR)}$$

where:

$$P_0 \ is \ the \ probability \ of \ the \ event \ in \ the \ reference \ group$$

On the other hand, when using negative binomial models, the exponentiated coefficient of a predictor variable yields a multiplicative factor indicating how the event occurrence rate is expected to change with a one-unit change in that variable. When the results of a negative binomial model are interpreted in a time-to-event context, the HR can be directly approximated by the exponential of the coefficient[3]:

$$HR = \exp(\beta)$$

[2] Zhang J, Yu KF. What's the relative risk? A method of correcting the odds ratio in cohort studies of common outcomes. JAMA. 1998;280:1690-1.

[3] Hilbe, JM. Negative binomial regression. Cambridge University Press. 2011
